# How a Retrotransposon Exploits the Plant's Heat Stress Response for Its Activation

**DOI:** 10.1371/journal.pgen.1004115

**Published:** 2014-01-30

**Authors:** Vladimir V. Cavrak, Nicole Lettner, Suraj Jamge, Agata Kosarewicz, Laura Maria Bayer, Ortrun Mittelsten Scheid

**Affiliations:** 1 Gregor Mendel Institute of Molecular Plant Biology, Austrian Academy of Sciences, Vienna, Austria; 2 Research Institute of Molecular Pathology, Vienna, Austria; and Institute of Molecular Biotechnology, Austrian Academy of Sciences, Vienna, Austria; National Institute of Genetics, Japan

## Abstract

Retrotransposons are major components of plant and animal genomes. They amplify by reverse transcription and reintegration into the host genome but their activity is usually epigenetically silenced. In plants, genomic copies of retrotransposons are typically associated with repressive chromatin modifications installed and maintained by RNA-directed DNA methylation. To escape this tight control, retrotransposons employ various strategies to avoid epigenetic silencing. Here we describe the mechanism developed by *ONSEN*, an LTR-copia type retrotransposon in *Arabidopsis thaliana*. *ONSEN* has acquired a heat-responsive element recognized by plant-derived heat stress defense factors, resulting in transcription and production of full length extrachromosomal DNA under elevated temperatures. Further, the *ONSEN* promoter is free of CG and CHG sites, and the reduction of DNA methylation at the CHH sites is not sufficient to activate the element. Since dividing cells have a more pronounced heat response, the extrachromosomal *ONSEN* DNA, capable of reintegrating into the genome, accumulates preferentially in the meristematic tissue of the shoot. The recruitment of a major plant heat shock transcription factor in periods of heat stress exploits the plant's heat stress response to achieve the transposon's activation, making it impossible for the host to respond appropriately to stress without losing control over the invader.

## Author Summary

Transposons are programmed to amplify within their host genomes. In defense, hosts have evolved mechanisms to impede transposon activation, often by epigenetic transcriptional silencing. A constant and likely unending arms race between host and invader has brought about different strategies to mutually counteract the tricks of the other. Several such strategies are combined in one transposon in the *Arabidopsis* genome. Its promoter is devoid of symmetric sites necessary for stable maintenance of repressive DNA methylation, and a reduction of methylation at the remaining cytosines does not activate the element. More sophisticated still: its promoter shares a sequence motif with heat stress-responsive plant genes and is recognized by a heat-induced plant transcription factor. Whenever the plants must activate their heat stress defense under high temperatures, the transposon is able to generate new extrachromosomal DNA copies that can potentially integrate into new sites of the genome. In addition, the heat response is especially strong in tissue with dividing cells, which form consequently the largest amount of extrachromosomal transposon copies. We see this as an example of a “wolf in sheep's clothing” strategy, whereby the transposon becomes visible as such only under specific stress conditions of its host.

## Introduction

Transposable elements (TEs) and their host organisms depend on each other for better or for worse. New TE insertions can give rise to deleterious mutations [1] or overall genetic instability [2], but they can also make a positive contribution to gene regulation and adaptation [3], [4]. Host organisms have developed mechanisms to reach a balance between both consequences by suppressing TE activity. Plants have evolved a complex regulatory network of epigenetic silencing that is effective for numerous different TEs. Silent elements are typically associated with high levels of DNA methylation at cytosines in every sequence context (^m^CG, ^m^CHG, ^m^CHH, where H stands for A, T or C), with methylation at lysine 9 of histone H3 (H3K9me2), and with the presence of 24 nt small interfering RNAs (siRNAs) that guide the RNA-directed DNA methylation (RdDM) machinery in a reinforcing loop, reviewed in [5], [6], [7]. Disruption of DNA methylation patterns can activate transposons, as, for instance, a null mutation of the *Arabidopsis* maintenance METHYLTRANSFERASE 1 (MET1) activates *EVADÉ* (*EVD*), a retrotransposon of the *ATCOPIA93* family that can amplify during sexual propagation of the mutant [8]. *EVADÉ* is also transcriptionally active in plants with hypomethylated DNA due to a lack of the chromatin-remodeling factor DECREASE IN DNA METHYLATION 1 (DDM1). *DDM1* mutants and many other plants lacking components of the RdDM pathway activate a specific but partially overlapping subset of TEs, including members of *ATCOPIA13*, *ATCOPIA21*, *ATGP3* retrotransposon families and *VANDAL21* and *CACTA* DNA transposons [9], [10], [11].

In addition to genetic interference, TEs can be activated by stress. In fact, this was already postulated by the discoverer of TEs [12] who also recognized the important role of these elements for gene regulation. Stress-induced transposon activation was later documented by molecular data in many different hosts, for instance the activation of the *Tnt1* element by pathogens in tobacco [13], [14], of the *ZmMI1* in maize and the *PAL/Tam3* in snapdragon by cold [15], [16], [17], or of the *CLCoi1* by wounding or salt stress in lemon [18]. More recently, a *Ty1/copia*-type long terminal repeat (LTR) retrotransposon family (*ATCOPIA78*) named *ONSEN* was found activated by heat stress in *Arabidopsis* [19], [20]. Surprisingly, without heat stress, *ONSEN* was not expressed in *ddm1* mutants [19] or other mutants lacking RdDM components, in contrast to most other known and potentially functional TEs in *Arabidopsis*. However, new *ONSEN* insertions were found in the progeny of heat-stressed plants deficient in small RNA production, and this retrotransposition appears to occur during flower development and before gametogenesis [21]. Higher activation in callus compared to vegetative tissue indicates a possible coupling to active cell cycling [22]. Transcription of *ONSEN*-related sequences after heat exposure was found in most species of the *Brassicaceae* [23], indicating a conserved mechanism of activation and control of their spreading.

Here, we provide insight into the initiation of *ONSEN* activation in *Arabidopsis*, quantifying transcription, and the formation of extrachromosomal DNA upon extended heat stress. We also determine which of the genomic copies become transcriptionally active. Although the LTR sequences representing the *ONSEN* promoter are methylated, reduction of the methylation is not sufficient to activate the element. Rather, the LTR has acquired a sequence that is recognized by the plant's heat-responsive transcription factors, thereby coupling *ONSEN* activation to an important stress defense. Even more cunningly, this response is most pronounced in regions comprising the meristematic zone, providing an enhanced chance for new *ONSEN* copies to enter the next generation.

## Results

### Transcriptional activation of *ONSEN* during heat stress is followed by efficient multiplication of extrachromosomal DNA copies

**Figure 1:**
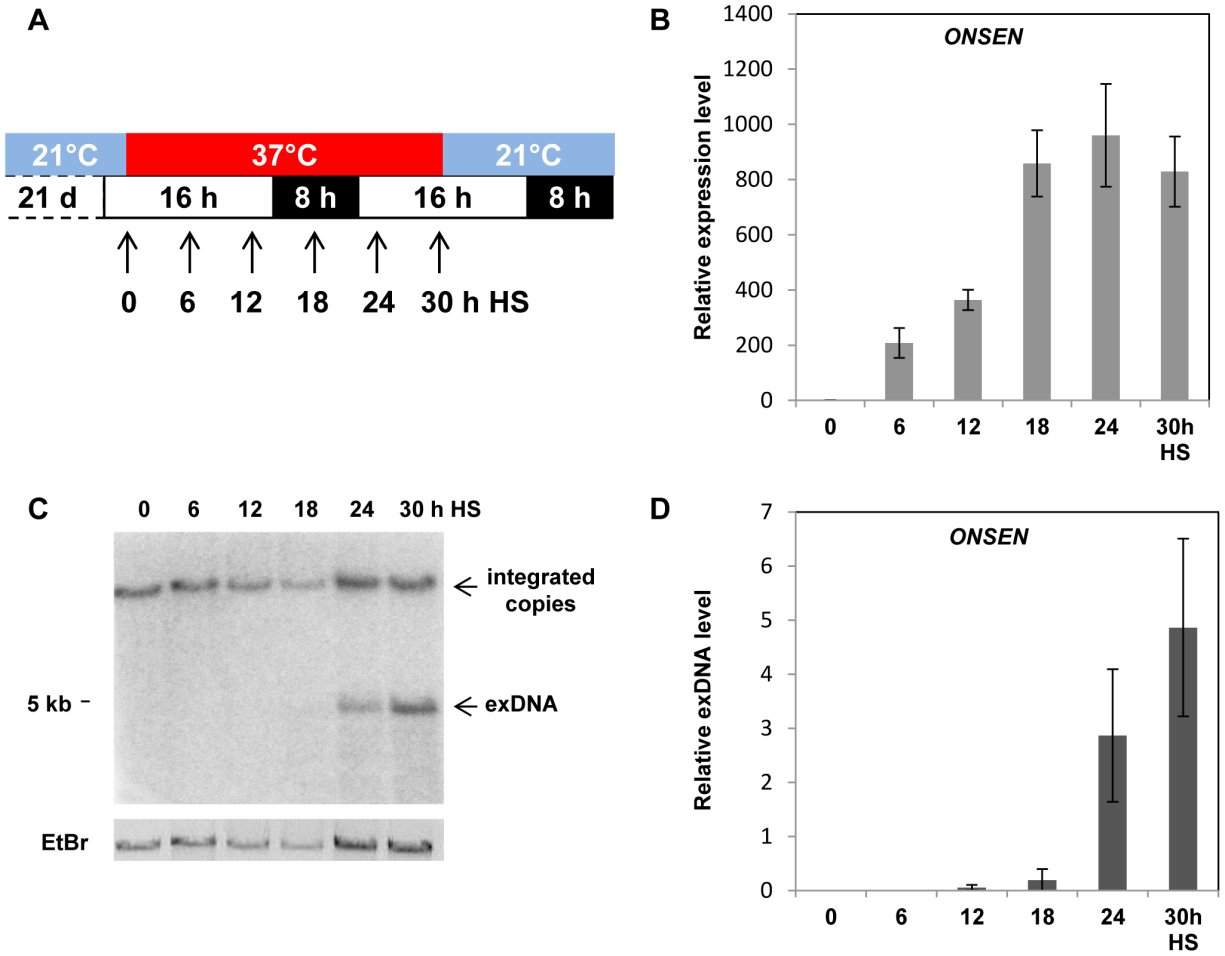
*ONSEN* is activated by heat stress. (**A**) Schematic representation of the experiments. White and black boxes represent light or darkness intervals; blue and red boxes represent periods of standard growth temperature or heat stress, arrows indicate sampling time points. (**B**) Relative amounts of *ONSEN* RNA determined by quantitative RT-PCR in three week-old wild type seedlings harvested as indicated in A. Bars represent the *ONSEN* transcripts in relation to that of AtSAND (equal level in all samples) and normalized to WT at 0 h HS. Error bars correspond to the s.e.m. (n = 3). (**C**) Southern blot analysis of undigested genomic DNA isolated from the same material as in B and hybridized to an *ONSEN*-specific probe. Upper and lower arrows indicate integrated and extrachromosomal *ONSEN* copies, respectively. The EtBr image indicates the loading of genomic DNA. (**D**) Quantification of *ONSEN* extrachromosomal DNA based on C. Bars represent the ratio between signal intensities of extrachromosomal and integrated copies determined by densitometry. Error bars correspond to the s.d. (n = 3).

*A. thaliana* grows mainly in zones with moderate climates. A temperature of 37°C represents acute and drastic heat stress for this plant, as shown by the quick transcriptional activation of many heat shock factors, [reviewed in 24]. The heat-induced transcriptional activation of retrotransposon *ONSEN* was also observed above a threshold of 37°C [19], [20], [22], though only after an extended exposure to heat [19], [20]. To determine the kinetics of *ONSEN* activation in more detail, we monitored the *ONSEN* expression at several time points during 30 h of heat stress (HS) treatment, keeping the regular long-day light regime (Fig. 1A). This treatment causes substantial growth arrest but is sublethal and allows recovery of the plants upon subsequent transfer to ambient temperature [19]. *ONSEN* RNA was first quantified by qRT-PCR 6 hours after the onset of heat stress, and its amount continued to increase to the highest level after 24 h, remaining high until 30 h, the end point of the stress treatment (Fig. 1B).

Following transcriptional activation, retrotransposons form linear extrachromosomal DNA copies along with circular by-products of replication; these are capable of reintegrating in the genome [25], [26], [27]. To determine the presence and amount of extrachromosomal *ONSEN* DNA, we performed Southern blot analysis with non-digested DNA from samples collected at the same time points as for the RNA. The single, high molecular weight band hybridizing to the *ONSEN* probe in the non-heat stressed samples corresponds to *ONSEN* copies integrated in the genomic DNA (gDNA). An additional band present in later heat stress samples indeed indicated extrachromosomal *ONSEN* DNA (exDNA), with a size of 5 kb corresponding to the expected full length of the linear element that is capable of reintegrating in the genome (Fig. 1C). To quantify the relative amount of exDNA, we calculated the relative intensity of hybridization signals between exDNA and gDNA in each sample, postulating a fixed number of integrated *ONSEN* copies. Small amounts of linear exDNA of *ONSEN* first appeared after 12 hours of heat stress. The maximum was achieved after 30 hours HS, corresponding to approximately five times more than integrated in the genome (Fig. 1D). Therefore, the formation of the linear extrachromosomal *ONSEN* DNA follows the transcriptional activation during heat stress after approximately 6 hours, likely reflecting the need for a threshold level of RNA and time to complete the reverse transcription. Once started, the process can produce many more additional copies than templates present in the genome.

### Most extrachromosomal DNA is derived from younger genomic copies

**Figure 2:**
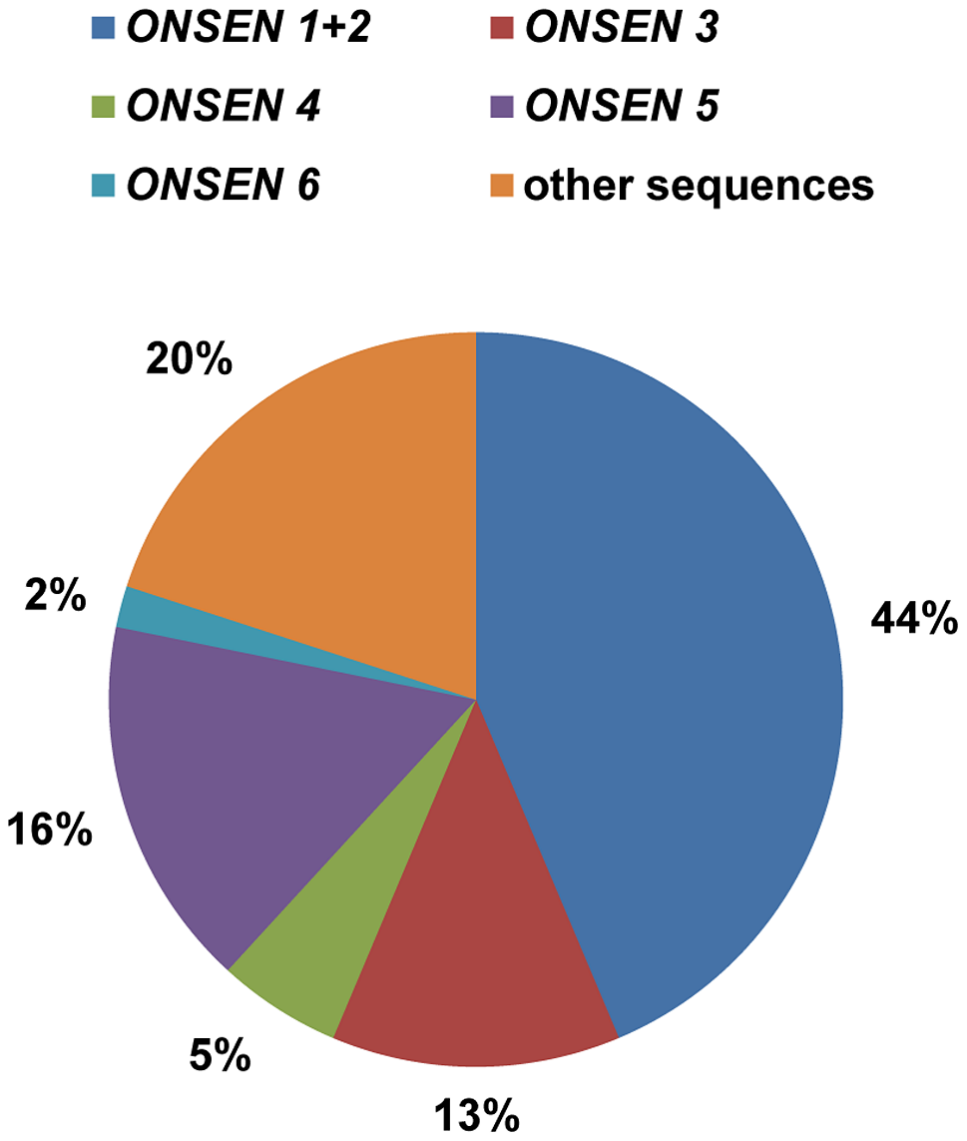
Several *ONSEN* genomic copies contribute to the extrachromosomal DNA. The pie chart indicates the ratio of extrachromosomal *ONSEN* sequences (n = 55) present after 30 h HS, distinguished by element-specific polymorphisms (color-coded). Polymorphisms are listed in Supplementary Table S1.

The family of *ONSEN* retrotransposons in the Col-0 reference genome consists of eight full length copies distributed over chromosomes 1, 3, and 5 (Supplementary Fig. S1A). To investigate whether all copies contribute to the pool of extrachromosomal DNA under heat stress, we performed a sequencing analysis of isolated exDNA, scoring for element-specific SNPs in the *ONSEN* coding region that distinguish seven out of eight genomic templates (Supplementary Fig. S1A, Supplementary Table S1). Out of 55 independent clones, 57% could be assigned to three elements (*ONSEN 1*: *At1g11265*; *ONSEN 2*: *At3g61330*; and *ONSEN 3: At5g13205*; Fig. 2) that form a subgroup with 100% identical 5′and 3′ LTRs, indicating their evolutionarily recent transposition [21]. Several other sequences (20%) contained additional and different SNPs, so not allowing an unambiguous assignment to genomic templates and supporting the notion that reverse transcription is an error-prone process [28].

In spite of the prevalence of the elements with perfect LTRs, three other *ONSEN* elements from the Col-0 genome contributed 23% to the pool of sequences, indicating that *ONSEN 4, 5 and 6* (*At1g58140*, *At1g48710* and *At3g59720*) are still functional and capable of forming extrachromosomal copies. In contrast, *ONSEN 7 and 8* (*At1g21945* and *At3g32415*) were not represented. Interestingly, these two copies are shared between most natural accessions of *Arabidopsis* [23], and they have acquired the largest number of polymorphisms.

### DNA demethylation of the LTR is not sufficient to activate *ONSEN* but favors its heat stress response

**Figure 3:**
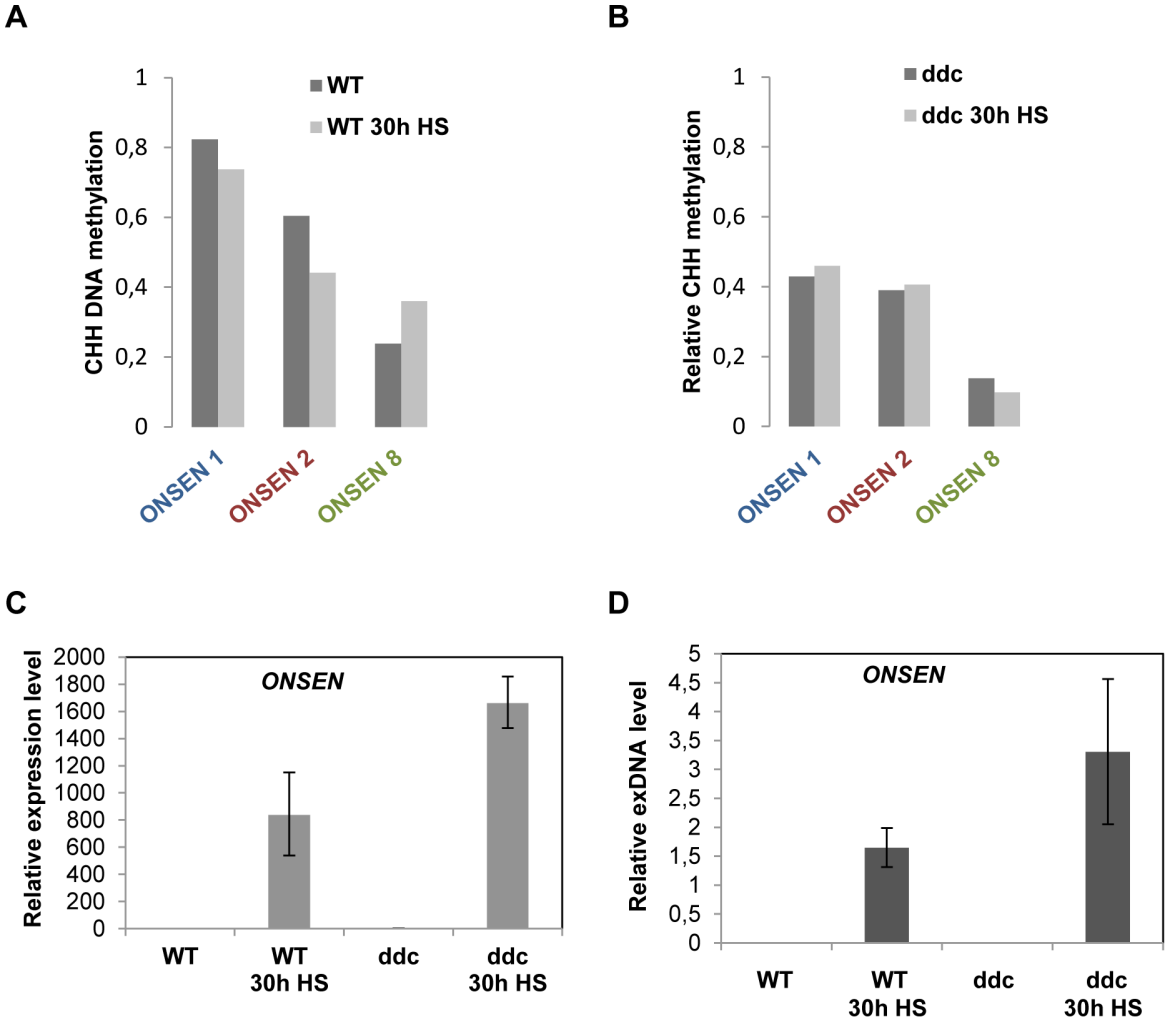
Loss of CHH DNA methylation at the promoter is not sufficient to activate the element but enhances the response to heat stress. (**A**) Total CHH methylation at LTRs of *ONSEN 1 and 2* (contributing the majority of exDNA) and *ONSEN 8* (not activated) in non-stressed and 30 h HS wild type plants. Only CHH positions shared between all three LTRs were considered. CHH methylation profiles along the LTRs and data for individual genomic copies are shown in Supplementary Fig. 2A–F and Supplementary Dataset S1. (**B**) Total CHH methylation of the same *ONSEN* LTRs in non-stressed and 30 h HS drm1/drm2/cmt3 triple mutant plants (ddc). Only CHH positions shared between all three LTRs were considered. CHH methylation profiles along the LTRs and data for individual genomic copies are shown in Supplementary Fig. 2G–L and Supplementary Dataset S1. (**C**) Relative amount of *ONSEN RNA* determined by quantitative RT-PCR in three week old wild type and ddc mutant plants, non-stressed and after 30 h HS. Bars represent the *ONSEN* transcripts in relation to that of *AtSAND* and normalized to WT at 0 h HS. Error bars correspond to the s.e.m. (n = 3). (**D**) Quantification of *ONSEN* extrachromosomal DNA from the same samples as in F. Bars represent the ratio between extrachromosomal and integrated copies determined by densitometry after Southern blot analysis. Error bars correspond to the s.d. (n = 3).

RdDM is the major pathway to restrict transposon mobility by installing DNA methylation, leading to a reinforcing loop that creates a heterochromatic environment. Most RdDM targets have multiple CHG and CHH sequences, which become modified by cooperative action of two different DNA methyltransferases. However, the LTR of active *ONSEN* copies contains only CHH sites (Supplementary Fig. S1B). To analyze the DNA methylation state at the 5′ LTRs of several *ONSEN* elements, we performed bisulfite conversion and sequencing before and after heat stress, distinguishing three genomic copies by specific primers in the flanking genomic regions (Supplementary Table S2). At ambient temperature, the two most recently transposed elements (*ONSEN 1* and *2*) showed relatively high CHH methylation profiles across their 5′LTRs (Fig. 3A, Supplementary Fig. S2A, B, and Supplementary Dataset S1). Strikingly, *ONSEN 8* that was not activated had substantially less methylation under normal growth conditions (Fig. 3A, Supplementary Fig. S2C). After heat stress, *ONSEN* 8 was more methylated throughout the 5′LTR, while *ONSEN 1* and *2* lost the modification at several positions (Fig. 3A, Supplementary Fig. S2D–F).

To investigate whether this erasure of DNA methylation had any impact on the level of *ONSEN* activation, we repeated RNA and exDNA quantification as well as DNA methylation analysis in the triple mutant ddc (*drm1/drm2/cmt3*), lacking RdDM-mediated methylation at CHH and CHG sites [29]. As expected, the levels of CHH methylation were significantly reduced at the *ONSEN* LTRs even in unstressed mutant plants (Fig. 3B, Supplementary Fig. S2G–I, and Supplementary Dataset S1). However, this was not sufficient to trigger any *ONSEN* transcription or exDNA formation (Fig. 3C, D). After heat stress, the already reduced CHH methylation in the triple mutant was hardly changed (Fig. 3B, Supplementary Fig. S2J–L and Supplementary Dataset S1), but the levels of *ONSEN* mRNA, as well as its extrachromosomal DNA, were significantly increased after heat stress compared to Col-0 wild type (Fig. 3C, D). Sequencing 47 clones of *ONSEN* extrachromosomal DNA from ddc mutant plants after heat stress revealed an even more dominant representation of activated *ONSEN 1* and *2* than in the wild type (Supplementary Fig. S3). Collectively, these results indicate that reduction of DNA methylation at the retrotransposon's promoter does not *per se* activate the element but can favor the activation of *ONSEN* upon heat exposure.

### *ONSEN* activation requires the plant's heat stress response pathway

The strong activation of *ONSEN* by heat stress conditions, together with the enhanced response in the methylation triple mutant, suggested the dependence on a heat-induced transcription factor whose action could be modulated by presence or absence of the DNA modification. Therefore, we analyzed the *ONSEN* promoter for *cis*-regulatory elements and found a heat response element (HRE) with the consensus sequence nTTCnnGAAn in the LTR of all elements (Supplementary Fig. S1C). HREs are bound by heat shock factors (HSFs), which form trimers and thereby induce expression of downstream target genes [30]. *Arabidopsis* has 21 heat shock factors [31], among which HSFA1 was identified as a main positive regulator in heat-responsive gene expression [32].

**Figure 4:**
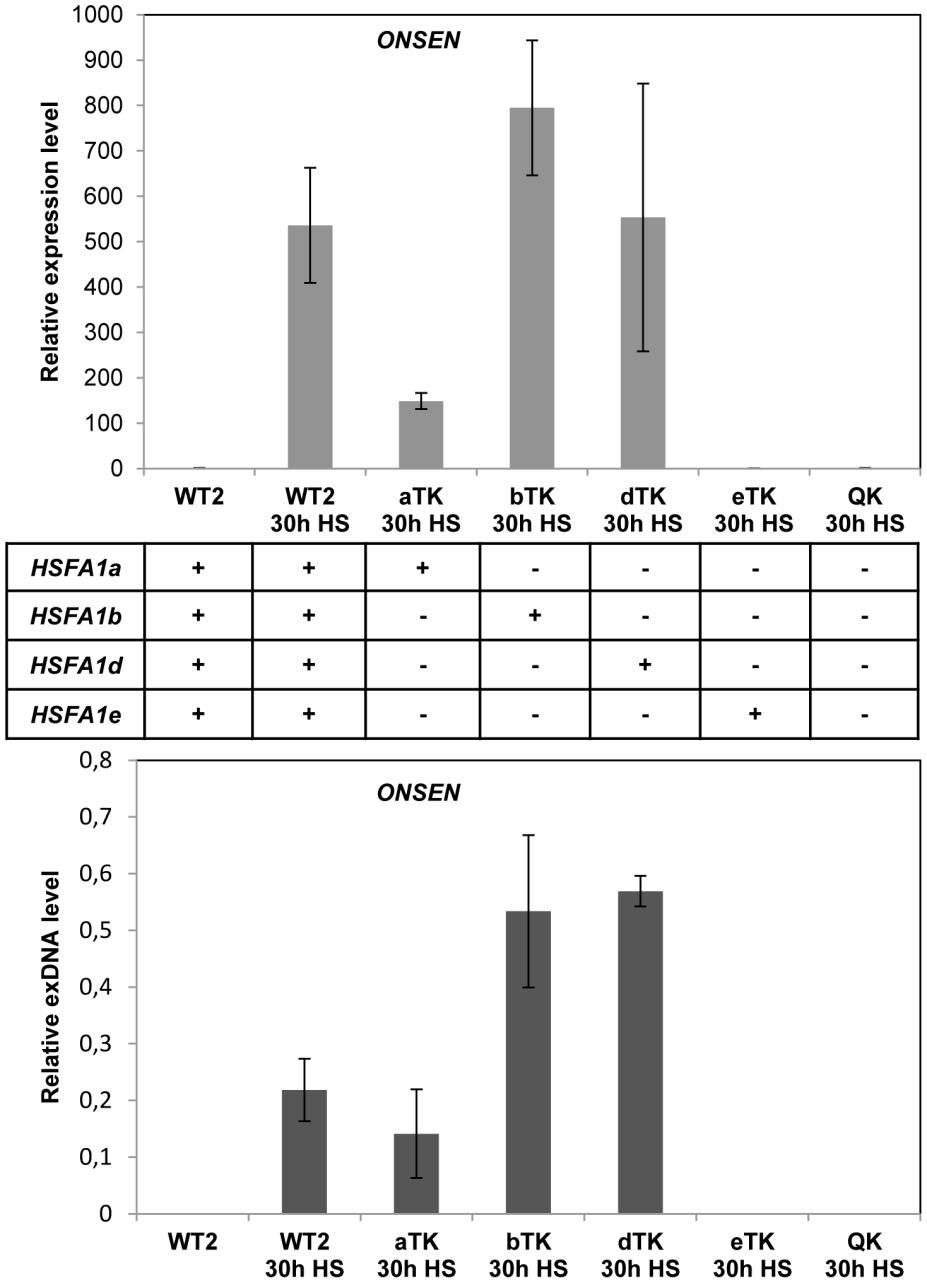
Heat shock factors are necessary for *ONSEN* activation by heat stress. Relative amounts of *ONSEN* RNA and extrachromosomal DNA prepared from three week-old, non-stressed or 30 h HS treated seedlings of wild type Wassilewskija (WT2, background of the mutants), triple or quadruple mutants lacking three or all functional HSFA1 proteins (A, B, D, E). RNA and exDNA were determined by quantitative RT-PCR and densitometry after Southern blot analysis as described before (n = 3).

To analyze whether HSFA1 mediates the transcriptional activation of the retrotransposon we determined *ONSEN* RNA and exDNA in *hsfa1* mutants. There are 4 genes for HSFA1-type proteins in *Arabidopsis* [a, b, d, e; 32] with partially redundant functions. We tested all different combinations of triple mutants along with the quadruple mutant (Fig. 4). HSFA1b or d were sufficient to activate *ONSEN* to comparable levels as in the wild type, and HSFA1a to a lower extent. In contrast, no *ONSEN* RNA or exDNA was formed after heat stress in the quadruple mutant or in plants with HSFA1e as the only functional HSFA1 factor. Therefore, either HSFA1a, or HSFA1b, or HSFA1d are necessary for heat-induced expression of *ONSEN*. However, they were unlikely candidates for direct transcriptional activators of *ONSEN*, since all three genes are constitutively expressed and proposed to initiate a cascade of heat stress-responsive genes only upon additional signals [32].

### *ONSEN* activation requires the plant's heat-induced transcription factor HSFA2

**Figure 5:**
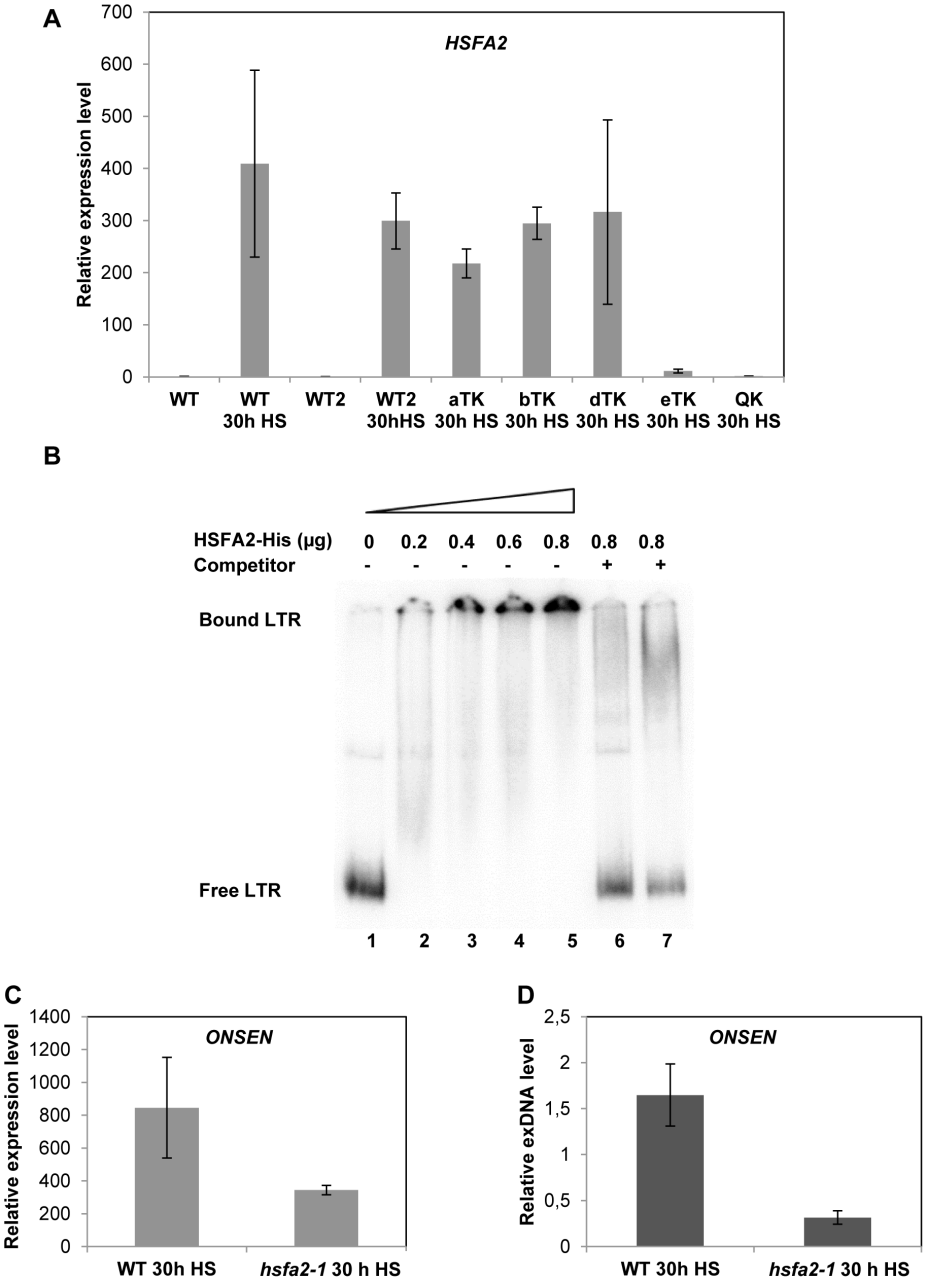
HSFA2 acts downstream of HSFA1 and is able to bind to the *ONSEN* LTR. (**A**) Relative amount of *HSFA2* RNA determined by quantitative RT-PCR in three week old WT (Col-0), WT2 (Ws), triple and quadruple *HSFA1* mutant seedlings. Bars represent the *HSFA2* transcript in relation to *AtSAND* and normalized to Col-0 WT at 0 h HS. Error bars correspond to the s.e.m. (n = 3). (**B**) Electrophoretic mobility shift assay (EMSA) with increasing amounts (lanes 1–5) of recombinant HSFA2 protein and constant amounts of radioactively labeled *ONSEN* LTR fragment derived from *ONSEN 1*. Competition by unlabeled DNA of the full length LTR in 50-fold excess (lane 6) or the fragment containing only the heat-responsive element (Supplementary Table S2) in a 200-fold excess (lane 7) demonstrate the binding specificity. (**C**) Relative amount of *ONSEN* RNA determined by quantitative RT-PCR in wild type and *hsfa2-1* mutant plants after 30 h HS. Bars represent the *ONSEN* transcripts in relation to that of *AtSAND* and normalized to WT at 0 h HS. Error bars correspond to the s.e.m. (n = 3). (**D**) Quantification of *ONSEN* extrachromosomal DNA from the same samples as in C. Bars represent the ratio between extrachromosomal and integrated copies determined by densitometry after Southern blot analysis. Error bars correspond to the s.d. (n = 3).

Transcripts of the *Arabidopsis* heat stress transcription factor *HSFA2* are not detectable at ambient temperatures, but the gene is most strongly and stably expressed under heat stress conditions [33]. The protein can be found in leaves after 3 hours of heat stress and is still present after 21 hours of recovery [34]. We quantified the *HSFA2* transcript under our heat stress conditions in wild type, *hsfa1* triple and quadruple mutants. Interestingly, *HSFA2* transcription showed the same dependence on the individual HSFA1 factors as *ONSEN*, hardly or not at all transcribed upon lack of HSFA1a,b,d or of all four factors (Fig. 5A). This suggested HSFA2 as a candidate for the heat stress factor necessary to activate *ONSEN*.

The HSFA2 protein has a highly conserved N-terminal DNA binding domain (DBD) that is required for its binding to HREs, and mutation within the domain or within the HRE block the binding [34] . To investigate whether HSFA2 would bind to the HRE in the *ONSEN* promoter we performed electrophoretic mobility shift assays (EMSAs) with recombinant *Arabidopsis* HSFA2 protein and the LTR DNA (Fig. 5B). Indeed, HSFA2 bound to the LTR probe in a concentration-dependent and specific manner: increasing amounts of the protein enhanced the shift (Fig. 5B, lanes 1–5), while pre-incubation with non-labeled LTR fragment inhibited the shift of the labeled probe (Fig. 5B, lane 6). The specificity of HSFA2 binding to the HRE in the LTR could be further supported by successful binding competition with an LTR fragment containing just 51 bp with the complete HRE (Fig. 5B, lane 7).

To further confirm the involvement of HSFA2 in transcriptional activation of *ONSEN* we quantified RNA and extrachromosomal DNA with and without heat stress in the *hsfa2-1* mutant (Fig. 5C, D). *ONSEN* activation was severely reduced in the mutant, although not to the same low level as in the *hsfa1a/b/d* triple or the *hsfa1a/b/d/e* quadruple mutants. This indicates that HSFA2 is a major, but probably not the only heat shock factor involved in the heat-induced activation of *ONSEN*. However, it is clear that the HRE in its promoter couples the retrotransposon to the heat response pathway of the plant, thereby exploiting an important defense mechanism of its host to prepare for its own amplification.

### The heat-responsive LTR of *ONSEN* is preferentially active in tissues with cell divisions

**Figure 6:**
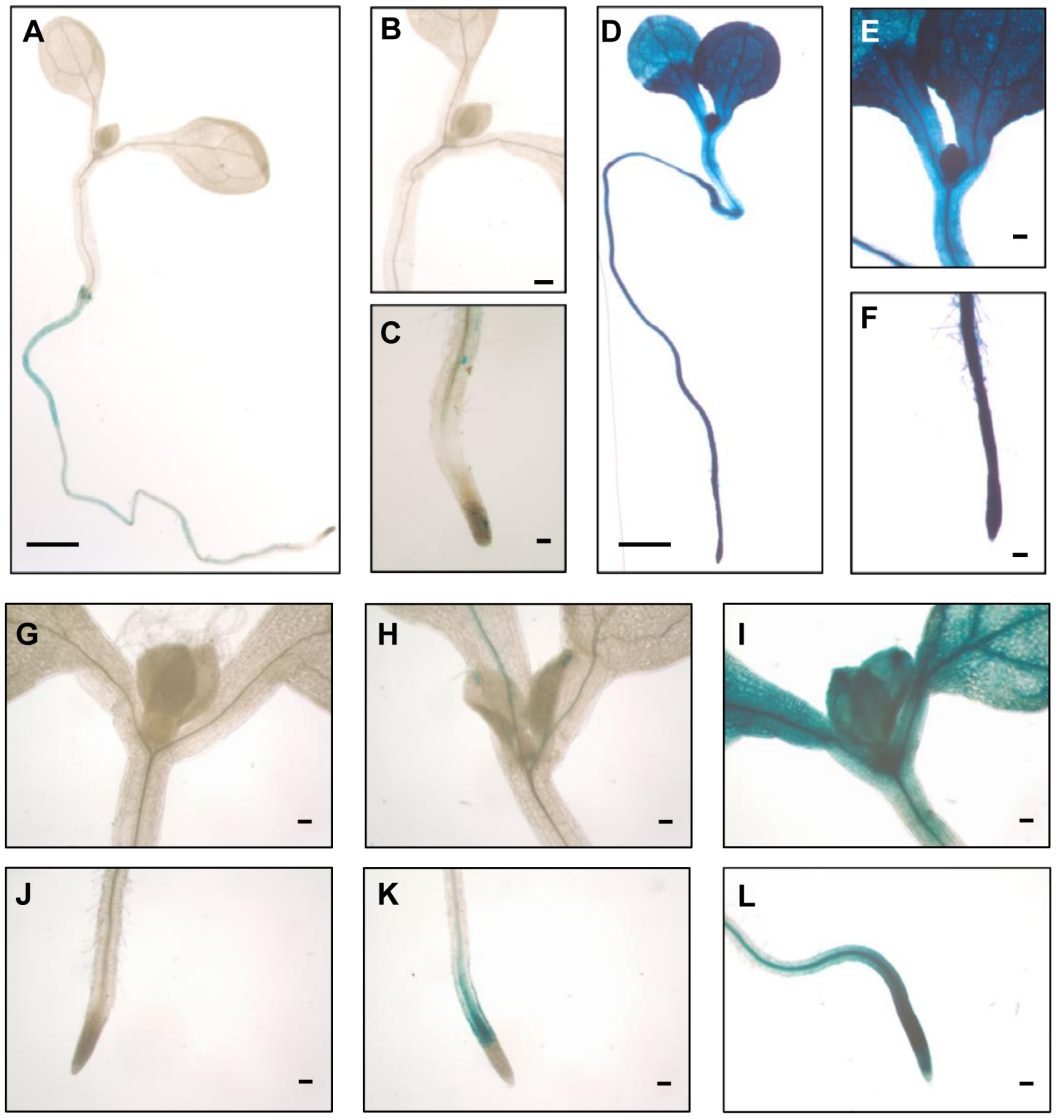
The *ONSEN* LTR is rapidly activated throughout the seedlings. Transcriptional activity of the *ONSEN* LTR fused to the GUS reporter gene in Col-0 wild type plants in one week-old seedling under non-stressed (**A–C**) and 30 h heat stress conditions (**D–F**). Details of shoot (**G–I**) and root (**J–L**) images of GUS stained seedlings after 30 min (**G, J**), 1 h (**H, K**) and 2 h heat stress (**I, L**). Scale bars represent 1 mm (**A** and **D**) or 0.1 mm (other panels).

Transgenic analysis of the *HSFA2* promoter activity by fusion with the *GUS* reporter gene had revealed very low activity under non-stress conditions but high expression under heat stress, in rosette leaves and even more in veins, root tips and root branching points [35]. We asked if the expression pattern of the *ONSEN* promoter would be similar and constructed a transgene consisting of the full 440 bp LTR upstream of a sequence encoding a *GUS-GFP* fusion protein (Supplementary Fig. S4A). This construct was introduced into wild type plants, and T4 plants homozygous for a single copy insert of the transgene analyzed for reporter gene expression. Under ambient temperatures, very low GUS activity was detectable in some root cells, while the 30 h heat stress treatment resulted in deep blue staining all over the plants (Fig. 6A–F). Expression starts already shortly after the beginning of the stress, as the first staining in leaf and root tips can be seen as early as 1 h, sometimes also at specific sites such as the leaf tips or emerging root branches, similar to the pattern seen for *HSFA2* (Supplementary Fig. S4B, C; [35]). Reporter gene expression later spreads rapidly (Fig. 6G–L). Live imaging of the GFP expression confirms the preferential promoter activity of the LTR in dividing tissues (Supplementary Fig. S4D, E). Correspondingly, the amount of extrachromosomal *ONSEN* DNA after heat exposure in meristem-enriched tissue exceeds by 8 times that in leaves (Supplementary Fig. S4F), thereby producing a high number of retrotransposon copies ready to reintegrate into the genome of cells that give rise to the next generation. This might represent an especially successful strategy of the element to exploit the plant's own indispensable stress protection mechanism to increase the probability of proliferation.

## Discussion

Transposons employ various strategies to gain replication advantage over their hosts in order to produce additional copies. In this study we show that *ONSEN* exploits a conserved stress defense response to initiate its amplification, in tissue that ultimately produces the germ line, and avoiding stable maintenance of DNA methylation.

Several mobile elements in many organisms are activated upon different environmental cues. Closest to the example described here is the heat responsiveness of Copia elements in Drosophila. While the data on the rate of transposition and their interpretation are controversial [reviewed in 36], there is unquestioned evidence for a correlation between heat-induced expression of a copia type retrotransposon and the presence of heat shock consensus sequences in the promoter [37]. However, in contrast to *ONSEN*, the Drosophila element is transcribed even without heat stress, the expression increase is faster but much less pronounced, and its promoter lacks at least three more copies of the nGAAn sequence required for high affinity interactions with HSFs [30]. Accumulation of DNA copies of the Drosophila element was not described and is unlikely, since a substantial amount of transcript is needed to generate sufficient Gag and Pol proteins and to serve as template for reverse transcription. In the case of the tobacco *Tto1* retrotransposon studied in a heterologous system, Gag protein and linear DNA molecules are synthetized in a ratio of 1400∶1 [38]. Therefore, *ONSEN* has been (more) successful in acquiring an efficient and specific heat-responsive regulatory element that allows production of extrachromosomal DNA copies by far exceeding the genomic copies.

Extended heat stress conditions can transiently release epigenetic regulation from several genes and transposons [19], [20], and most of these do not have a heat responsive element in their promoter. Some transposons respond equally well to other types of stress [39]. High copy number repeats like *TSI* and the *GUS* transgene are effectively activated also in an *hsfa2* mutant [19]. The requirement of the HSFA factors suggests that the pronounced *ONSEN* activation is not just a consequence of a general loss of epigenetic control as for the other elements. At least one of the three genes *HSFA1a, b or d* is necessary to generate *ONSEN* transcript and extrachromosomal DNA, and the triple mutant shows severely impaired resistance and viability under heat stress conditions [40]. Expression of HSFA2, the dominant heat shock factor downstream of the HSFA1 complex in *Arabidopsis* [33] able to bind to the HRE of several target genes [34], is almost completely eliminated in the *hsfa1a/b/d* triple mutant upon heat exposure, like *ONSEN*. The specific binding of the HSFA2 protein to the HRE in the *ONSEN* LTR *in vitro* demonstrates that *ONSEN* can recruit this factor, thereby “masking” itself as a heat shock gene and coupling its transcriptional activation to the plants' heat stress response. Reduced but not completely abolished *ONSEN* transcription in the *hsfa2* mutant indicates that it can also acquire other factors from the partially redundant heat-related transcription factor family.

HSFA2 protein is found in leaves, stem, flowers, green siliques and roots of heat-stressed plants [34], and the *GUS* reporter with the *ONSEN* LTR shows a similar ubiquitous expression after longer heat exposure. However, the early and strong expression of the ectopic *ONSEN GUS* fusion construct in tissue with dividing cells resembles the more pronounced expression in the root meristems and root branch points of the *HSFA2* promoter [35]. Although an analysis of microarray data for transposon expression under ambient temperatures in different *Arabidopsis* tissue did not provide evidence for a preferential expression in dividing tissue [41], *ONSEN* activation upon heat stress was elevated in undifferentiated callus [22], and several mobile elements in maize are preferentially expressed in the shoot apical meristem [42]. This and other evidence has led to the hypothesis of developmental relaxation of TE silencing (DRTS) and suggested that transposons could amplify preferentially in tissues or cells that undergo epigenetic reprogramming in the course of developmental processes [43]. Accordingly, reintegration of *ONSEN* into new locations occurred frequently in somatic cells during flower formation, although only in epigenetically compromised mutant plants [21]. Therefore, exploiting the higher activity of the heat stress defense in highly dividing tissue for a preferential accumulation of extrachromosomal *ONSEN* copies might be another optimization strategy of the element to achieve a higher probability of transmission to the next generation. What appears as developmental relaxation might, in some cases at least, be a similarly sophisticated adaptation.

In addition to coupling itself to the plants' stress response and preferentially producing amplicons where propagation chances are high, the sequence of the *ONSEN* promoter might represent another refinement. Its LTR has a GC content of 28%, not far from the average of 34.7% in the *Arabidopsis* genome [44], but it does not contain any C in palindromic arrangement between the strands. This is in contrast to LTRs of similar length and composition of other elements that are under the control of the RdDM pathway: *EVADÉ* [8] (*AtCopia93*, 406 bp, 37% GC, 5 CG and 6 CHG) and *solo-LTR* [45] (376 bp, 27%GC, 2 CG, 3 CHG). The absence of CG in the *ONSEN* promoter circumvents maintenance methylation by MET1, and the lack of any CHG site also precludes modification by CMT3. This, in consequence, interferes with the reinforcing loop in which the histone methyltransferase KRYPTONITE (KYP) installs H3K9me2 [46], thereby strongly impeding the formation of heterochromatin at the *ONSEN* LTR. The CHH sites in the promoter can only be modified by CMT2 [47] or RdDM, two synergistic pathways that are responsible for substantial methylation of transposable elements in *Arabidopsis*. While ONSEN was not amplified in non-stressed ddc mutant plants, the activation after heat stress was more pronounced compared to wild type. Since the lack of the RdDM-related methyltransferases DRM1 and DRM2 reduced, but did not totally erase DNA methylation at ONSEN LTRs. the residual CHH methylation by CMT2 could potentially also contribute to the responsiveness of the promoter. However, lack of CMT2 is also not sufficient to completely eliminate CHH methylation at the ONSEN LTRs (Assaf Zemach and Daniel Zilberman, personal communication). This is only obtained in plants lacking both chromatin remodelers DDM1/DRD1 that support CMT2 and RdDM-mediated methylation [47]. However, even upon complete loss of CHH methylation in the double mutant *ddm1*/*drd1*, there is very weak activation of ONSEN in non-stressed plants (Assaf Zemach and Daniel Zilberman, personal communication). A more pronounced response of ONSEN to heat stress after partial demethylation may simply indicate better accessibility, as suggested for other regions in *Arabidopsis* [48].

In spite of the multiple levels at which *ONSEN* has optimized its transcription and production of extrachromosomal copies, it is an element with only a limited copy number in the *Arabidopsis thaliana* reference genome, and although other accessions have variable copy numbers [23], we do not know of any ecotype with a substantially larger number of inserts. Therefore, integration of extrachromosomal DNA copies into the plant genome seems to be a limiting factor, although rapid propagation of *ONSEN* was observed in the progeny of plants compromised in the RdDM pathway [21]. Restricting the chances for successful retrotransposon integration might indicate one efficient counter defense of the plants, the rapid mutation of already integrated copies another. Since retrotransposons usually remain in the genome and LTRs are identical upon insertion, an evolutionary history of degeneration like this can in fact be read from their sequence. The extrachromosomal *ONSEN* DNA copies were mainly produced from elements with 100% identical LTRs. These evolutionarily young elements are found in very few of the 95 *Arabidopsis* accessions tested [23], whereas elements not represented in the extrachromosomal fraction have acquired a large number of polymorphisms and are shared between most accessions. In addition to the eight recognizable full length copies, the reference genome has more than 10 sequences annotated as incomplete *Copia78* = *ONSEN* (TAIR database), remnants that indicate previous invasion but sequence degeneration of the element.

The tandem repeat array of the heat-responsive LTRs at the 5′ and 3′ end of *ONSEN* render any downstream sequence potentially transcribed upon heat stress, and a new insertion indeed conferred heat responsiveness to a neighboring gene [21]. Our finding that this is exerted by integration of the plants' target site for a heat shock transcription factor into the LTR lets us speculate that this is another example of co-evolution between the genomes of host and TE: activation of the element in times of stress might allow the element to propagate but provides at the same time some benefit by rendering additional plant genes stress-responsive, thereby generating genetic diversity as a premise for selection. It resembles other copia-like retrotransposon insertions in *Citrus* that convey cold induction of the transcription factor *Ruby* controlling anthocyanin production. This results in the intensive coloration of blood oranges [49] and is just one example that stress-induced transposon-mediated control of plant genes might also be of interest for plant breeders and agriculture.

On the one hand, the acquisition of the plants' heat responsive element by *ONSEN* masks the retrotransposon as familiar, corresponding to the “wolf-in-sheep's-clothing” strategy [50], and represents a particularly intriguing molecular parasitism in which it is impossible for the plant to respond appropriately to heat stress without the risk of retrotransposon amplification. On the other hand, plants might occasionally benefit from this strategy if their own regulatory element gets moved around upon stress into new insertion sites where it might prove useful. Evolution has the last word.

## Materials and Methods

### Plant material and growth conditions

The *hsfa2-1* [51] and *drm1 drm2 cmt3* triple mutant (ddc) [29], [52] in Col-0 background were previously described and obtained from the NASC stock center. *hsfa2* has a T-DNA insertion in At2g26150 (SALK_008978). *cmt3-11*, *drm1-2* and *drm2-2* are T-DNA insertion mutants in AT1G69770 (SALK 148381), At5g15380 (SALK 021316), and At5g14620 (SALK 150863), respectively. Also the *hsfa1* mutants were previously described [32], [53]. *hsfa1a* (former *hsf1-tt1*) has an insertion in At4g17750, *hsfa1b* (former *hsf3-tt1*) in At5g16820, *hsfa1d-1* in At1g32330 (SAIL_410_E01) and *hsfa1e* in At3g02990 (SALK_094943). Triple (aTK, bTK, dTK, eTK) and quadruple (QK) mutants of *HSFA1* generated in Col-0 and Wassilewskija background were a kind gift from Yee-yung Charng.

Plants were sown on germination medium and grown *in vitro* at 21°C under long-day conditions (16 h light/8 h dark) for 21 days prior to heat stress. For heat stress treatments (HS), plants were transferred to 37°C for 30 h (standard HS conditions) or shorter periods as indicated, starting 3 h after beginning of the light period. GFP and GUS images were taken on 7 day-old seedlings after standard heat stress conditions. Meristem-enriched tissue was prepared from 21 day-old seedlings by manual dissection of shoot tips smaller than 2 mm.

### RNA analysis

Total RNA was extracted from whole seedlings using the RNeasy Plant Mini Kit (Qiagen). An additional DNase treatment was performed to remove all extrachromosomal *ONSEN* DNA in the heat stress samples, prior to cDNA synthesis with random hexamer primers and the RevertAid M-MuLV Reverse Transcriptase (Thermo Scientific).

All qRT-PCR analyses were performed using the Sensi Mix SYBR & Fluorescin Kit (Bioline) and iQ5 equipment (Biorad). Each data point is based on nine PCR reactions deriving from three biological replicates. Expression values were normalized to AtSAND (At2g28390), documented to have equal expression levels under all tested conditions [54], [55].

### Quantification of extrachromosomal DNA

Genomic DNA was isolated from whole seedlings, leaves or meristem-enriched tissue using the Illustra DNA Extraction Kit Phytopure (GE Healthcare). Total DNA was separated on 0.8% agarose gel, depurinated for 10 min in 250 mM HCl, denatured in 0.5 M NaOH and 1.5 M NaCl for 30 min, and neutralized in 0.5 M Tris, 1.5 M NaCl and 1 mM EDTA at pH 7.2 for 2×15 min. DNA was blotted onto Hybond N+ membranes (Amersham) with 20× SSC, washed and UV-crosslinked with a Stratalinker (Stratagene).

Hybridization was performed as described [56]. An *ONSEN*-specific probe (see Supplementary Table 2) was radioactively labelled with 50 µCi of dCT-α-^32^P (Amersham) using the Rediprime II Random Prime Labelling System (GE Healthcare) and purified via G50 Probequant (Amersham) columns. Signals were detected using Phosphorimager screens (Amersham) and scanned by a Molecular Imager FX (Biorad). Densitometric quantification was performed using Image Lab software on three biological replicates.

### Sequencing extrachromosomal DNA

Undigested DNA prepared from whole seedlings was run on a 0.8% agarose gel, and a region around the size range of 5 kb, corresponding to the full length of linear *ONSEN* copies, was cut out. DNA was purified using the QIAquick Gel Extraction Kit (Qiagen). After a PCR amplification step, using *ONSEN*-SNPs F and R primers (Supplementary Table S2), the PCR product was purified with the MinElute Gel Extraction Kit (Qiagen) and ligated using the InsTAclone PCR Cloning Kit (Thermo Scientific). Individual clones were Sanger-sequenced.

### DNA methylation analyses

Bisulfite conversion of *BamHI* digested genomic DNA was performed using the Epitect Kit (Qiagen). Purified DNA after conversion was amplified and cloned for Sanger sequencing. Primers are listed in Supplementary Table S2. For each analysis, at least 13 independent clones were Sanger-sequenced. Sequencing data were visualized and the methylation percentage was calculated for each cytosine position using CyMate [57].

### Electrophoretic mobility shift assay

The full length of the *HSFA2* ORF was amplified by PCR from cDNA (see Supplementary Table S2), cloned in frame with the C-terminal 6× His-tag using *NdeI* and *XhoI* sites in the pET-24B vector. The construct was transformed into *E. coli* strain BL21(RIL) (Novagene) for expression. The bacterially expressed HSFA2-His fusion protein was purified with HisTrap 5 ml columns (GE Healthcare) and ÄKTA purifier (GE Healthcare) and used for EMSA. EMSA was performed as described previously [58]. The LTR sequence was amplified by PCR (see Supplementary Table S2) and the fragment gel-purified prior to labeling. For the competitor assay, complementary oligonucleotides (Supplementary Table S2) were annealed to generate double-stranded DNA. All probes were end-labeled using T4 PNK and (γ-^32^P)ATP and purified with G50 Probequant columns (Amersham). The binding assay was carried out in a buffer containing 10 mM Tris (pH 7.5), 1 mM EDTA, 0.1 M KCl, 0.1 mM DTT, 5% vol/vol glycerol and 0.01 mg/ml BSA. The binding reaction was incubated at RT for 30 min. The reaction mixtures were separated on 5% non-denaturating polyacrylamide gels in 0.5× TBE buffer at 140 V for 2 h, and the gels were exposed to Phosphoimager screens (Amersham) and scanned by a Molecular Imager FX (Biorad).

### Transgene construction and transformation

The 440 bp LTR sequence of *ONSEN 1* was cloned into the pENTR2B vector (Invitrogen) using *BamHI* and *EcoRV* restriction sites and subsequently into the pBGWFS7 binary vector [59] using the Gateway LR Clonase II Enzyme Mix (Invitrogen). The transgene (Supplementary Figure S4) was introduced into *Agrobacterium* strain AGL1, which was then used to transform Col-0 plants by floral dipping [60]. After selection of primary transformants by selection with Basta, plants were grown and allowed to self-pollinate. Plants homozygous for a single copy of the transgene (confirmed by Southern blot analysis) were selected and amplified. All experiments were done with plants of the T4 generation.

### GUS staining

GUS histochemical staining was performed as described [61].

## Supporting Information

Dataset S1 Bisulfite sequencing data from individual genomic copies.
(PDF)

Figure S1 (**A**) DNA alignment of all 8 *ONSEN* copies in the Col-0 reference genome. LTRs are indicated in green. The position of the Southern blot probe, the region analyzed for SNPs and the qPCR amplicon are indicated as black boxes. (**B**) Distribution of potential methylation sites in the LTRs of *ONSEN 1* and *2*. Note that all cytosines are in the CHH context (green triangles); CG sites or CHG sites are absent. (**C**) DNA alignment of all 5′LTRs of the 8 *ONSEN* copies in the Col-0 reference genome. Red arrows indicate heat responsive elements (HRE). nGAAn boxes in these HREs are highlighted with grey arrows.
(PDF)

Figure S2 CHH methylation profiles along the LTRs of *ONSEN 1* (blue, A,D,G.J) and *2* (red, B,E,H,K) contributing the majority of exDNA and *ONSEN 8* (green, C,F,I,L, not activated), in non-stressed (A–C, G–I) and 30 h HS (D–F, J–L) wild type (A–F) and ddc triple mutant (G–L) plants, according to bisulfite sequencing analysis. Only CHH positions shared between all three LTRs were considered. Sequencing data from individual clones are shown as Supplementary Dataset S1.
(PDF)

Figure S3 Pie chart indicating the ratio of extrachromosomal *ONSEN* sequences (n = 47) present after 30 h HS in the triple methylation mutant ddc, distinguished by element-specific polymorphisms (color-coded). Polymorphisms are listed in Supplementary Table S1.
(PDF)

Figure S4 (**A**) Schematic representation of the reporter construct with ONSEN promoter (LTR) and visible markers EGFP and GUS in translational fusion. Restriction enzyme EcoRV and Gateway recombination sequence attB2 refer to cloning sites. GUS staining of one week-old seedlings with the GUS reporter gene under control of the *ONSEN* LTR after 1 h (**B**) and 2 h heat stress (**C**). Shoot (**D**) and root (**E**) images of one week-old seedlings with the GFP reporter gene under control of the *ONSEN* LTR after 30 h HS. Scale bars represent 0.1 mm. (**F**) Quantification of *ONSEN* extrachromosomal DNA in dissected shoot tips and leaves of three week-old, non-stressed or 30 h HS WT Col-0. Bars represent the ratio between extrachromosomal and integrated copies determined by densitometry after Southern blot analysis. Error bars correspond to the s.d. (n = 2).
(PDF)

Table S1 Polymorphisms between *ONSEN* copies in accession Col-0.
(PDF)

Table S2 Oligonucleotide sequences.
(PDF)
