# Supplementary material for: How a Retrotransposon Exploits the Plant's Heat Stress Response for Its Activation
Source: PLoS Genet. 2014 Jan 30;10(1):e1004115. doi: 10.1371/journal.pgen.1004115 (PMC3907296; doi:10.1371/journal.pgen.1004115)

ONSEN 1 wt mock

CyMATE (c) 2007, 2008

Methylation overview of 'convertedColM.afa'

Class 1: ● me ○ not me

Class 2: ■ me □ not me

Class 3: ▶ me ▷ not me

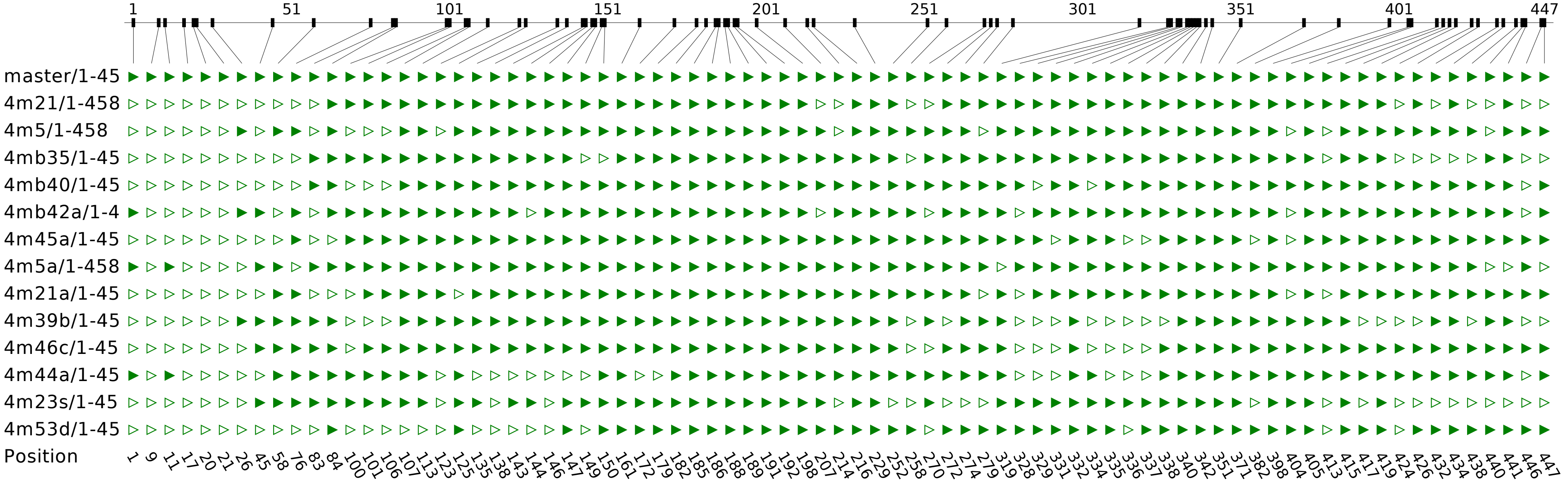

CyMATE (c) 2007, 2008
Methylation overview of 'convertedColM.afa'

Class 1:	● me	○ not me
Class 2:	■ me	□ not me
Class 3:	▶ me	▷ not me

448

	449
master/1-45	▶
4m21/1-458	▶
4m5/1-458	▶
4mb35/1-45	▶
4mb40/1-45	▶
4mb42a/1-4	▶
4m45a/1-45	▶
4m5a/1-458	▶
4m21a/1-45	▶
4m39b/1-45	▶
4m46c/1-45	▶
4m44a/1-45	▶
4m23s/1-45	▶
4m53d/1-45	▶
Position	449

ONSEN 1 wt HS

CyMATE (c) 2007, 2008
Methylation overview of 'converted Col HS.afa'

Class 1: ● me ○ not me
Class 2: ■ me □ not me
Class 3: ▶ me ▷ not me

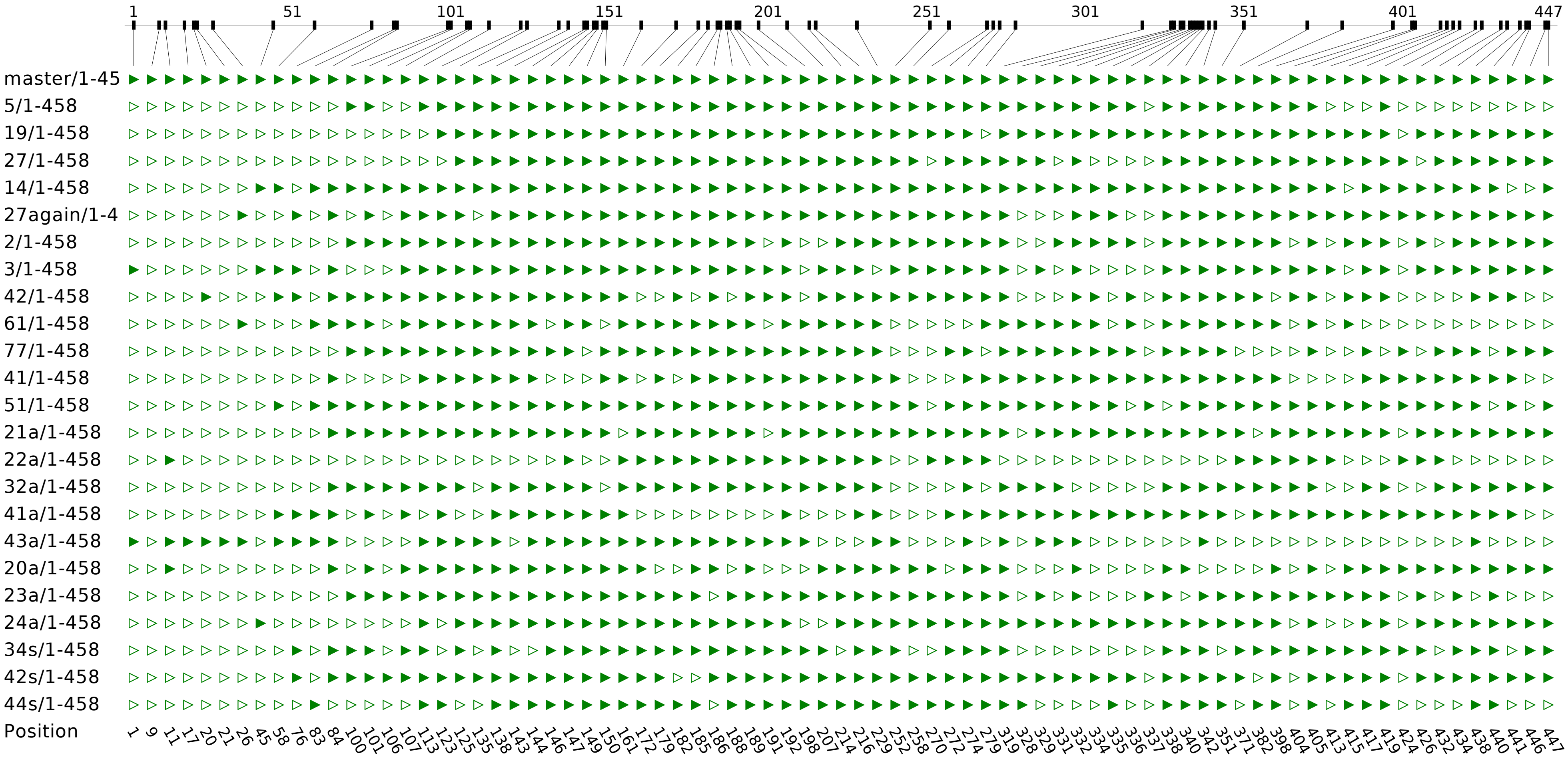

CyMATE (c) 2007, 2008
Methylation overview of 'converted Col HS.afa'

Class 1:	● me	○ not me
Class 2:	■ me	□ not me
Class 3:	▶ me	▷ not me

448

	449
master/1-45	▶
5/1-458	▶
19/1-458	▶
27/1-458	▶
14/1-458	▶
27again/1-4	▶
2/1-458	▶
3/1-458	▶
42/1-458	▶
61/1-458	▶
77/1-458	▶
41/1-458	▶
51/1-458	▶
21a/1-458	▶
22a/1-458	▶
32a/1-458	▶
41a/1-458	▶
43a/1-458	▷
20a/1-458	▶
23a/1-458	▶
24a/1-458	▶
34s/1-458	▶
42s/1-458	▶
44s/1-458	▶
Position	449

ONSEN 1 ddc mock

CyMATE (c) 2007, 2008

Methylation overview of 'converteddddcM.afa'

Class 1: ● me ○ not me

Class 2: ■ me □ not me

Class 3: ▶ me ▷ not me

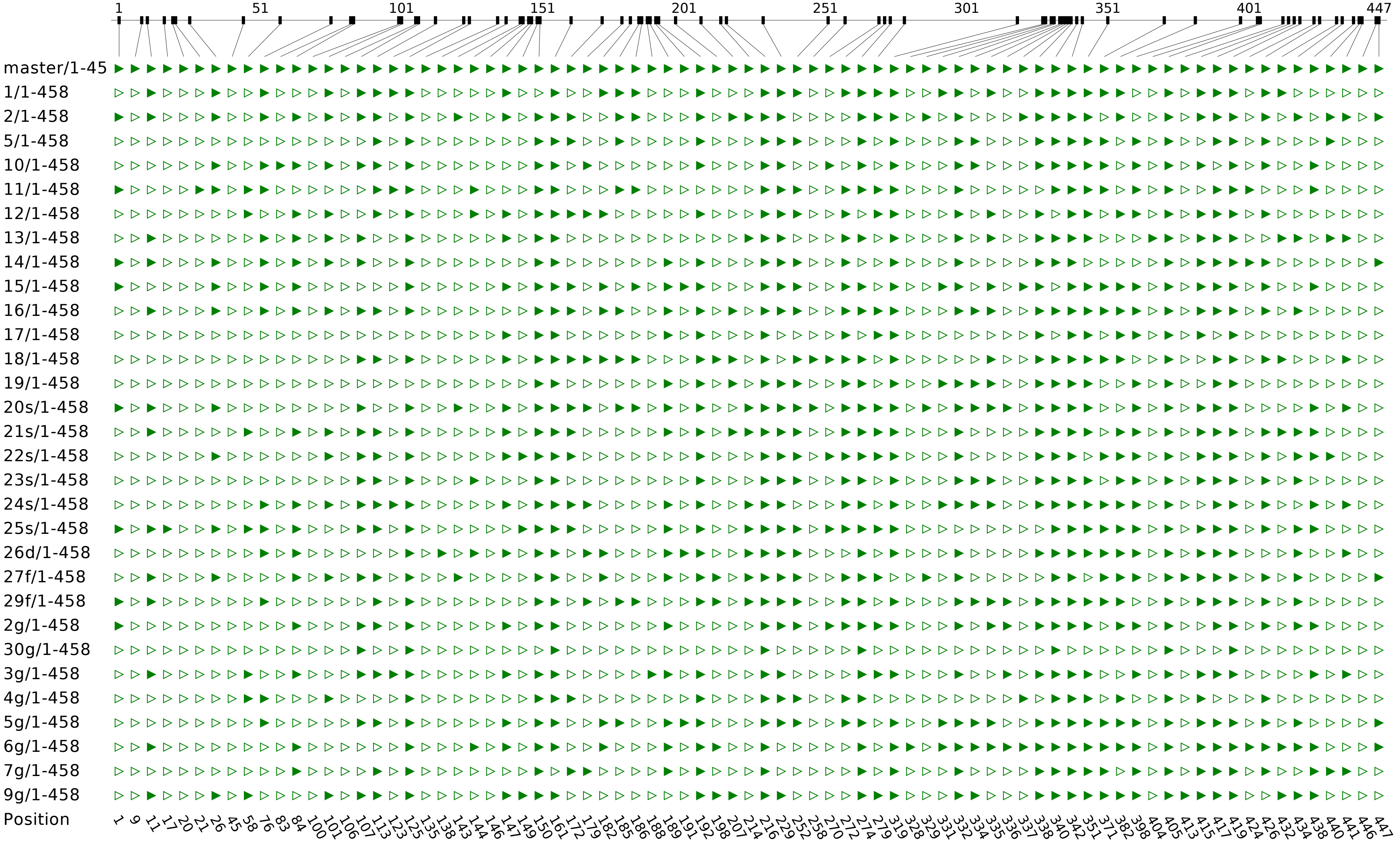

CyMATE (c) 2007, 2008
Methylation overview of 'converteddddcM.afa'

Class 1:	● me	○ not me
Class 2:	■ me	□ not me
Class 3:	▶ me	▷ not me

	448
master/1-45	▶
1/1-458	▶
2/1-458	▶
5/1-458	▶
10/1-458	▶
11/1-458	▶
12/1-458	▶
13/1-458	▶
14/1-458	▶
15/1-458	▶
16/1-458	▶
17/1-458	▶
18/1-458	▶
19/1-458	▶
20s/1-458	▶
21s/1-458	▶
22s/1-458	▶
23s/1-458	▶
24s/1-458	▶
25s/1-458	▷
26d/1-458	▶
27f/1-458	▶
29f/1-458	▶
2g/1-458	▶
30g/1-458	▶
3g/1-458	▶
4g/1-458	▶
5g/1-458	▶
6g/1-458	▶
7g/1-458	▶
9g/1-458	▶
Position	449

ONSEN 1 ddc HS

CyMATE (c) 2007, 2008
Methylation overview of 'converteddddcHS.afa'

Class 1: ● me ○ not me
Class 2: ■ me □ not me
Class 3: ▶ me ▷ not me

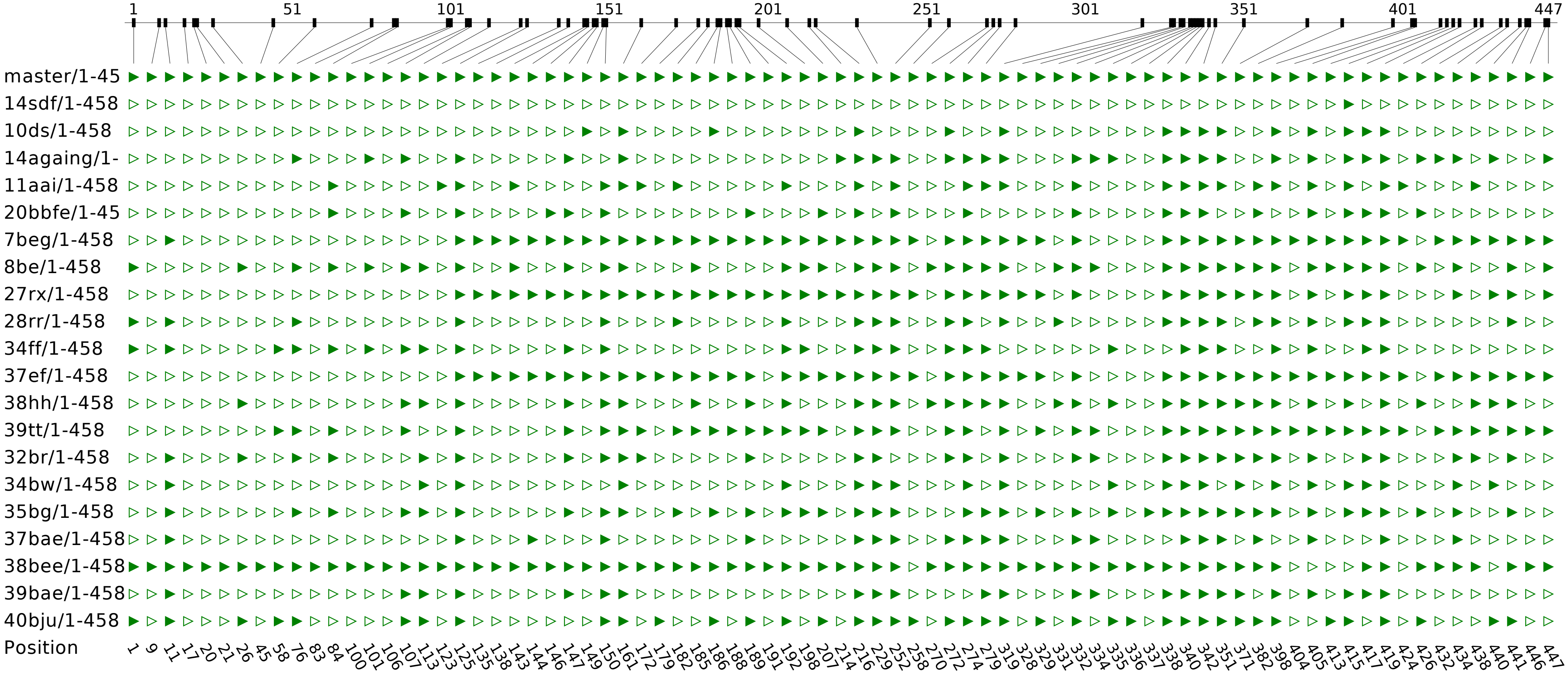

CyMATE (c) 2007, 2008
Methylation overview of 'converteddcHS.afa'

Class 1:	● me	○ not me
Class 2:	■ me	□ not me
Class 3:	▶ me	▷ not me

448

master/1-45 ▶
14sdf/1-458 ▷
10ds/1-458 ▶
14againg/1- ▶
11aai/1-458 ▶
20bbfe/1-45 ▶
7beg/1-458 ▶
8be/1-458 ▶
27rx/1-458 ▶
28rr/1-458 ▶
34ff/1-458 ▶
37ef/1-458 ▶
38hh/1-458 ▶
39tt/1-458 ▶
32br/1-458 ▶
34bw/1-458 ▶
35bg/1-458 ▶
37bae/1-458 ▷
38bee/1-458 ▶
39bae/1-458 ▶
40bju/1-458 ▶
Position 449

ONSEN 2 wt mock

CyMATE (c) 2007, 2008
Methylation overview of 'convertedColM.afa'

Class 1: ● me ○ not me
Class 2: ■ me □ not me
Class 3: ▶ me ▷ not me

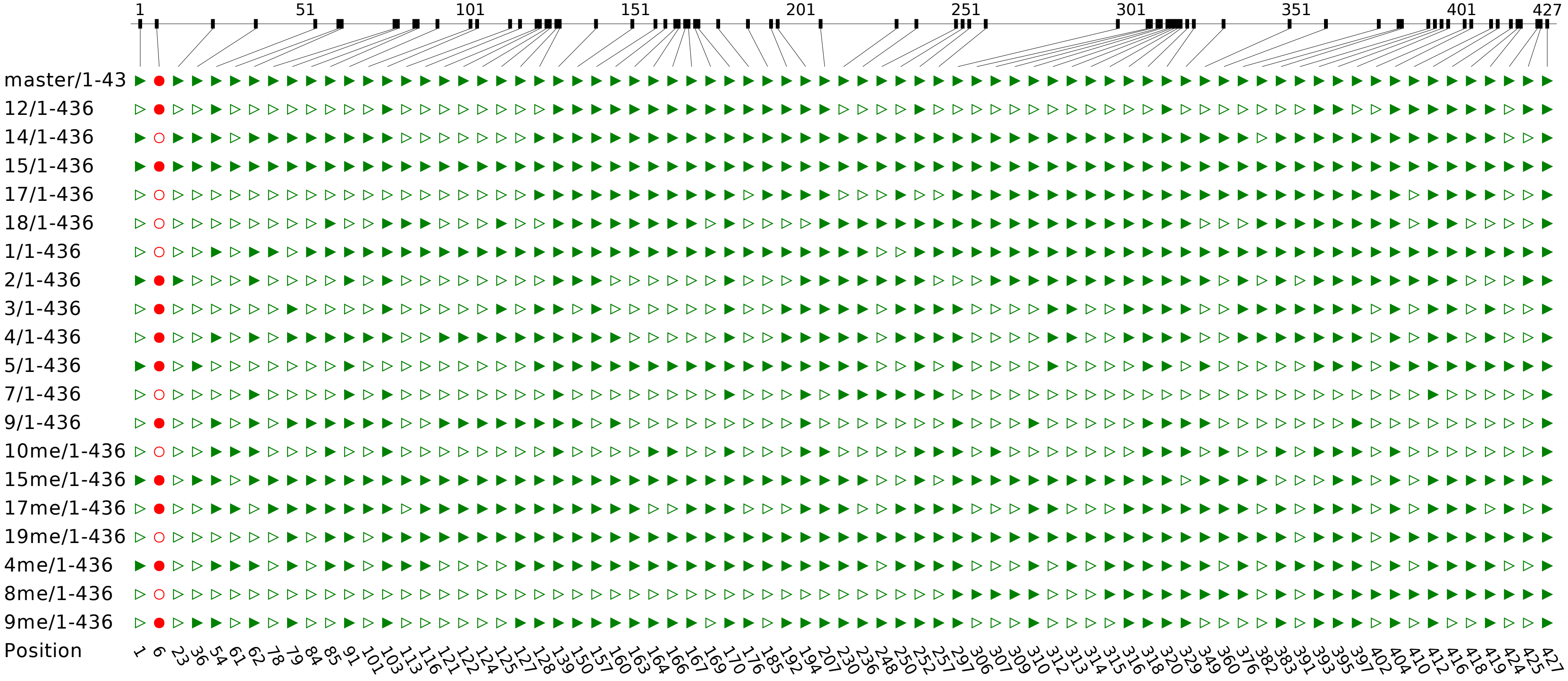

ONSEN 2 wt HS

CyMATE (c) 2007, 2008
Methylation overview of 'convertedColHS.afa'

Class 1: ● me ○ not me
Class 2: ■ me □ not me
Class 3: ▶ me ▷ not me

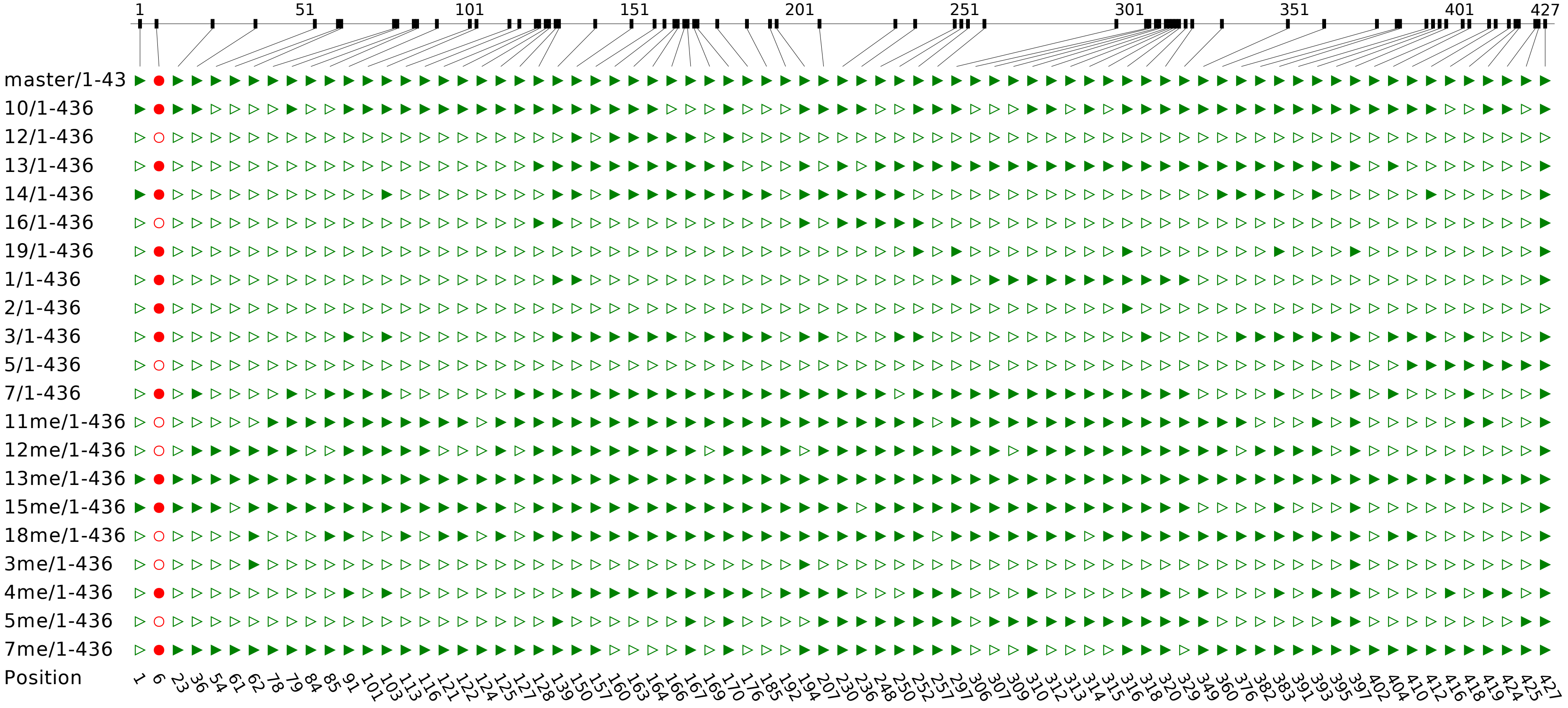

ONSEN 2 ddc mock

CyMATE (c) 2007, 2008
Methylation overview of 'converteddddcM.afa'

Class 1: ● me ○ not me
Class 2: ■ me □ not me
Class 3: ▶ me ▷ not me

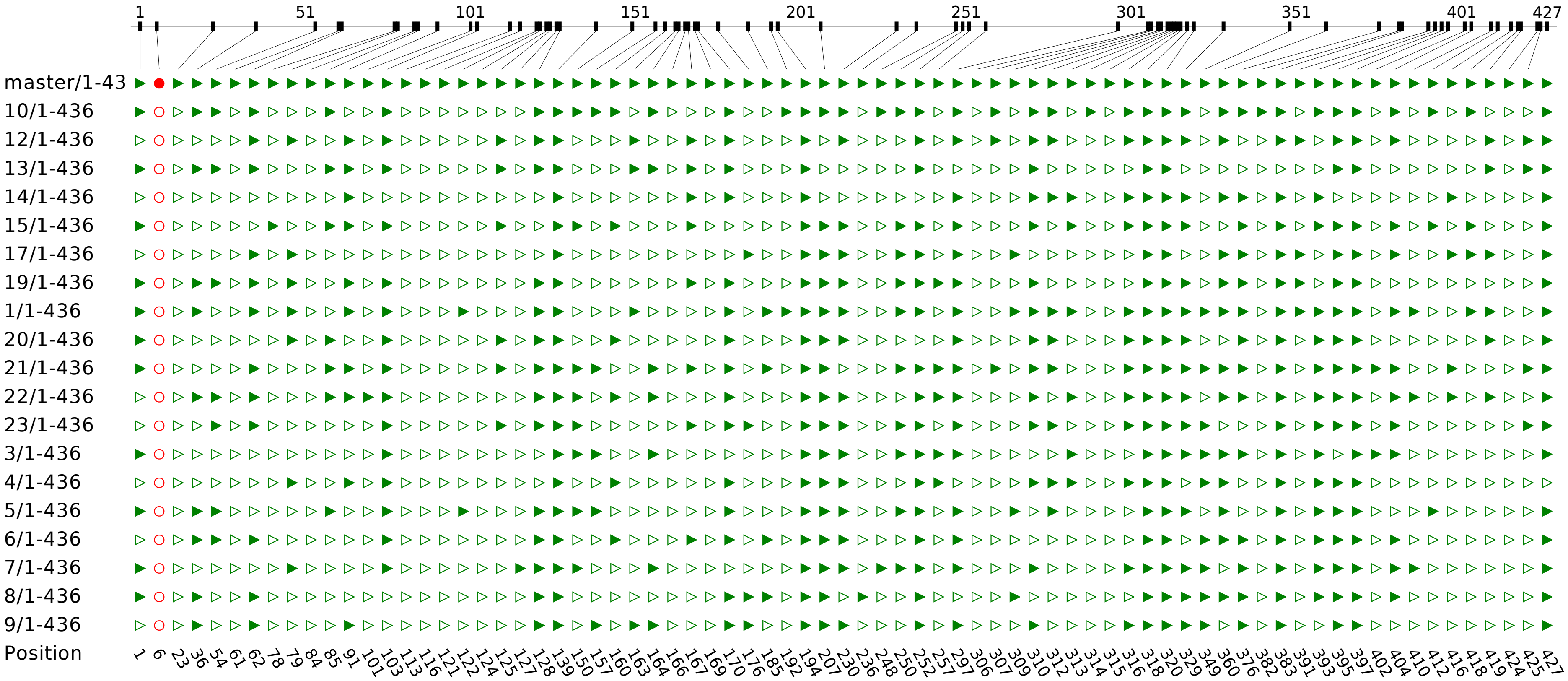

ONSEN 2 ddc HS

CyMATE (c) 2007, 2008
Methylation overview of 'convertedddcHS.afa'

Class 1:	●	me	○	not me
Class 2:	■	me	□	not me
Class 3:	▶	me	▷	not me

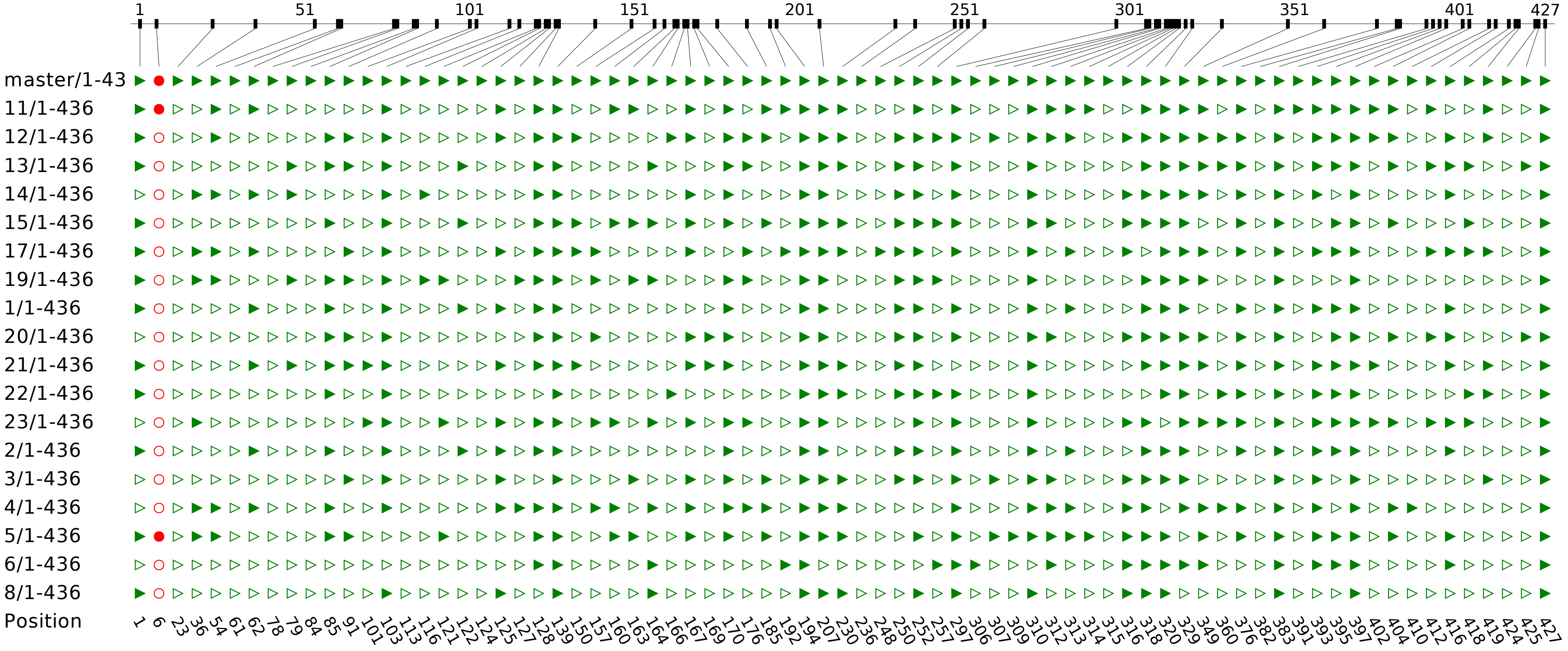

ONSEN 8 wt mock

CyMATE (c) 2007, 2008

Methylation overview of 'convertedCol M.afa'

Class 1: me / not me

Class 2: me / not me

Class 3: me / not me

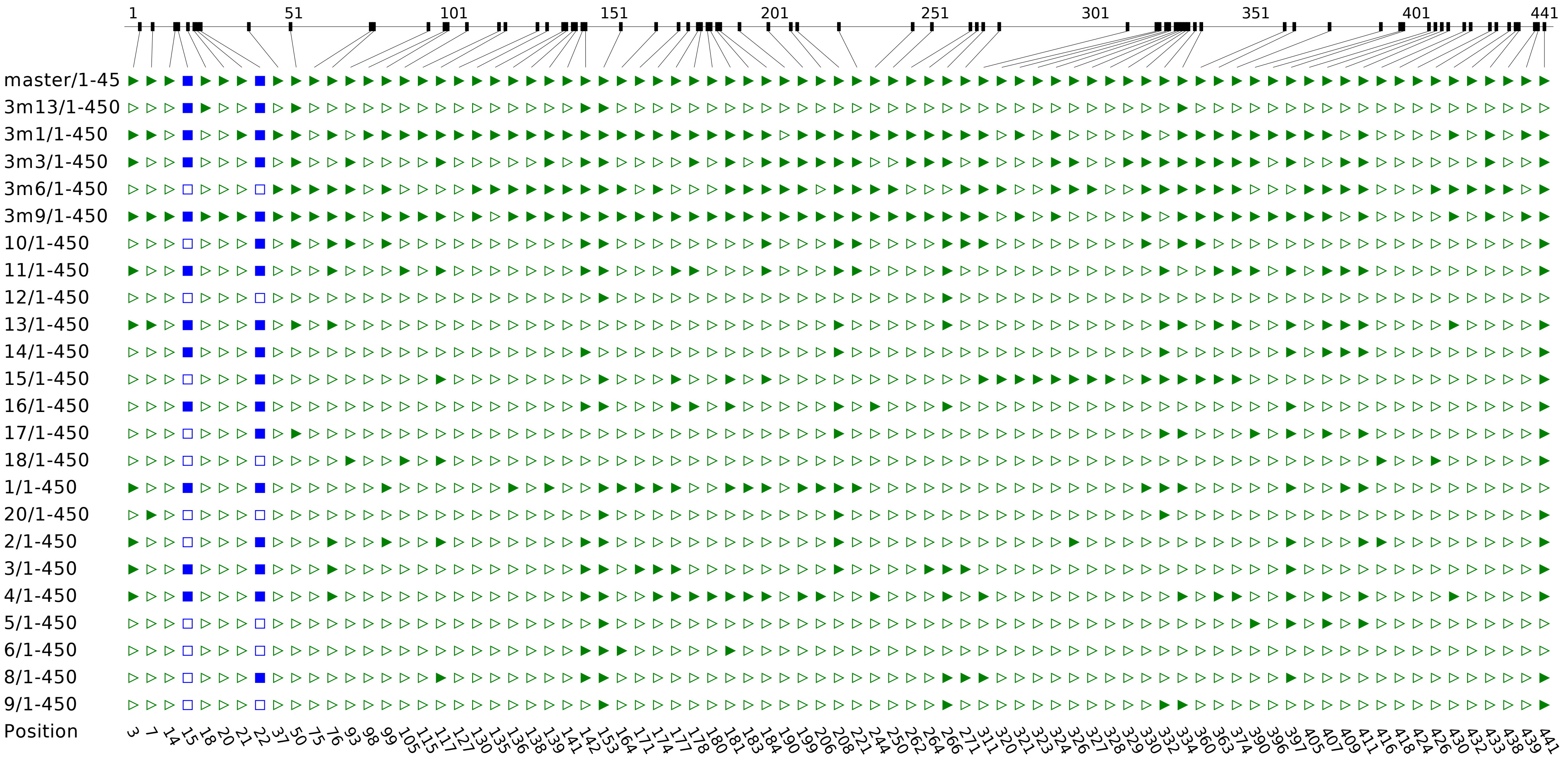

ONSEN 8 wt HS

CyMATE (c) 2007, 2008

Methylation overview of 'convertedColHS.afa'

Class 1: ● me ○ not me

Class 2: ■ me □ not me

Class 3: ▶ me ▷ not me

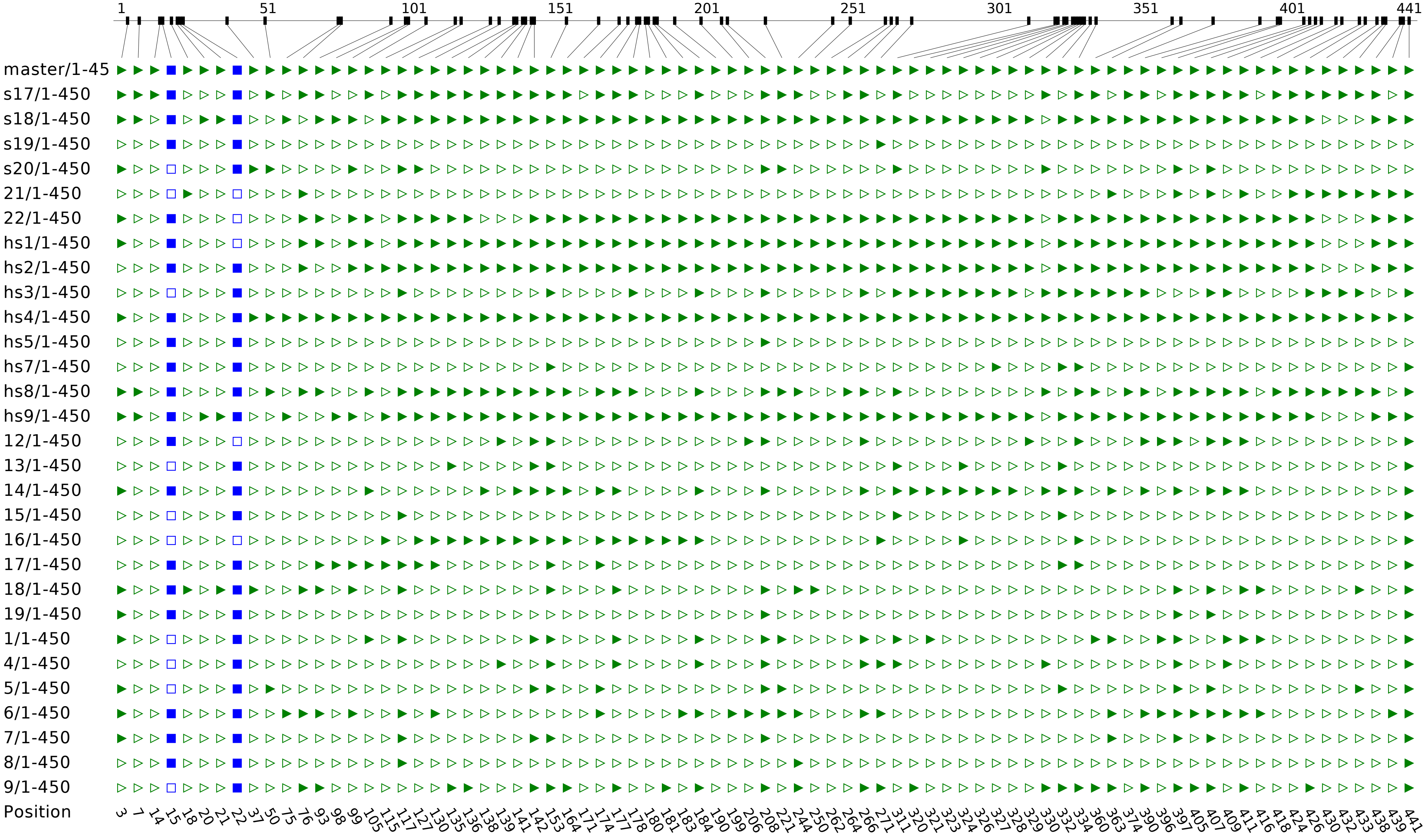

ONSEN 8 ddc mock

CyMATE (c) 2007, 2008
Methylation overview of 'converteddddcM.afa'

Class 1: ● me ○ not me
Class 2: ■ me □ not me
Class 3: ▶ me ▷ not me

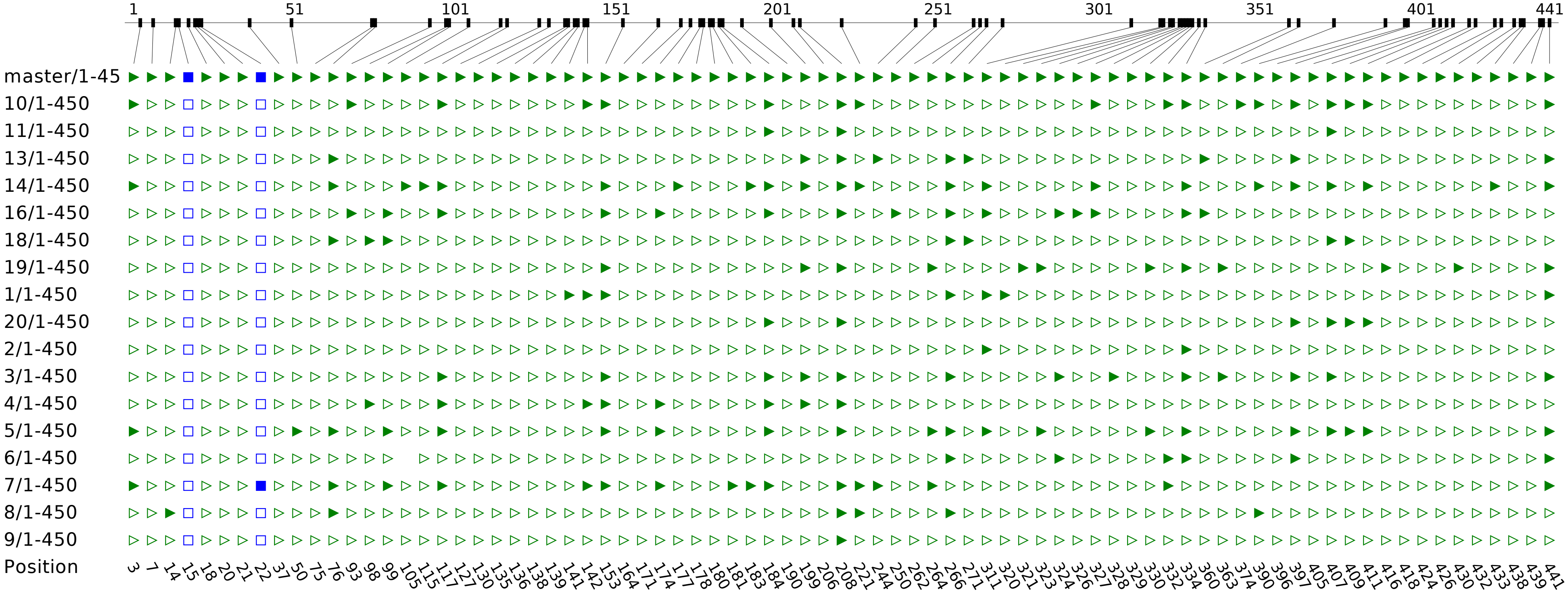

ONSEN 8 ddc HS

CyMATE (c) 2007, 2008
Methylation overview of 'convertedddcHS.afa'

Class 1: ● me ○ not me
Class 2: ■ me □ not me
Class 3: ▶ me ▷ not me

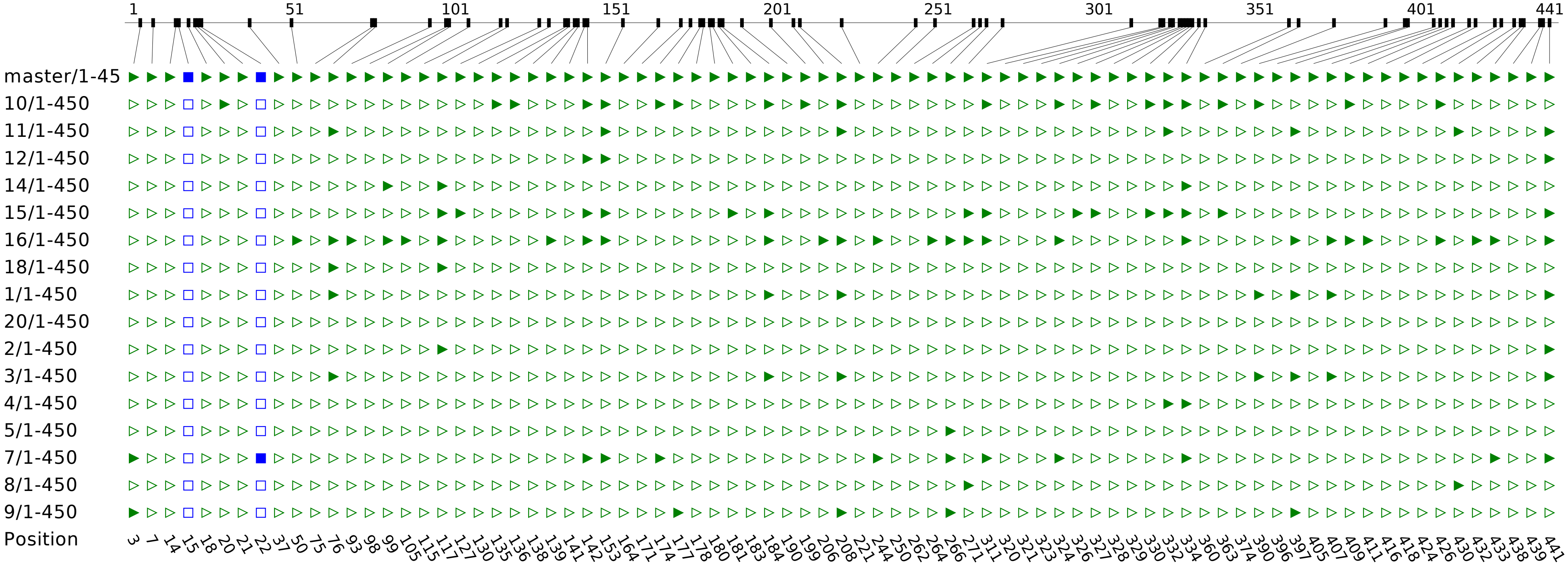

Supplement: Dataset S1 — Bisulfite sequencing data from individual genomic copies. (PDF) [file pgen.1004115.s001.pdf]
