# Supplementary figures and images for: How a Retrotransposon Exploits the Plant's Heat Stress Response for Its Activation
Source: PLoS Genet. 2014 Jan 30;10(1):e1004115. doi: 10.1371/journal.pgen.1004115 (PMC3907296; doi:10.1371/journal.pgen.1004115)

**A**

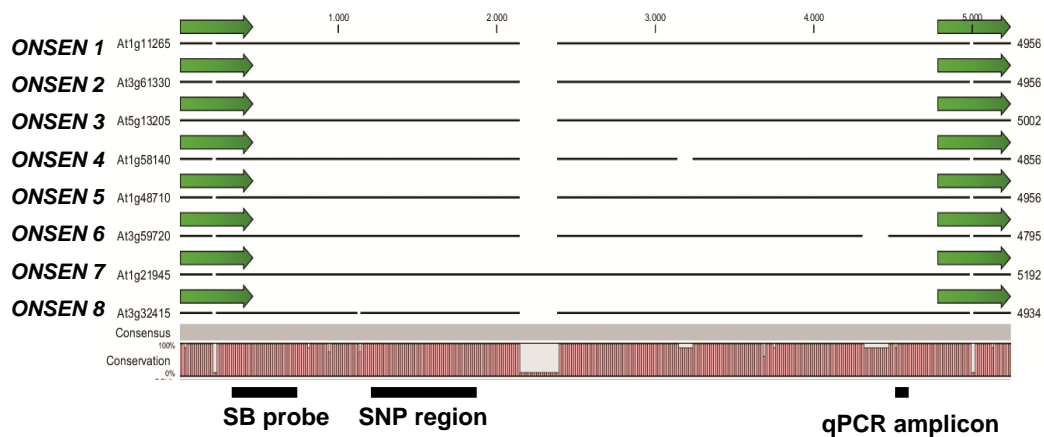

**B**

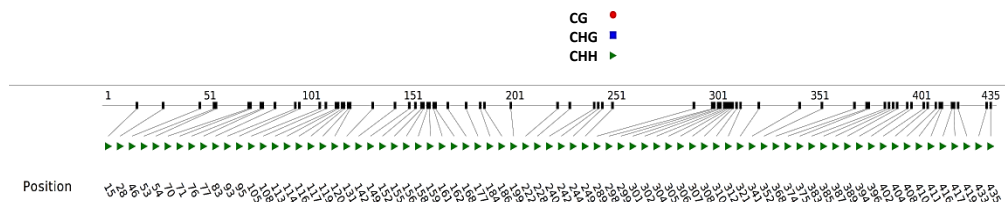

**C**

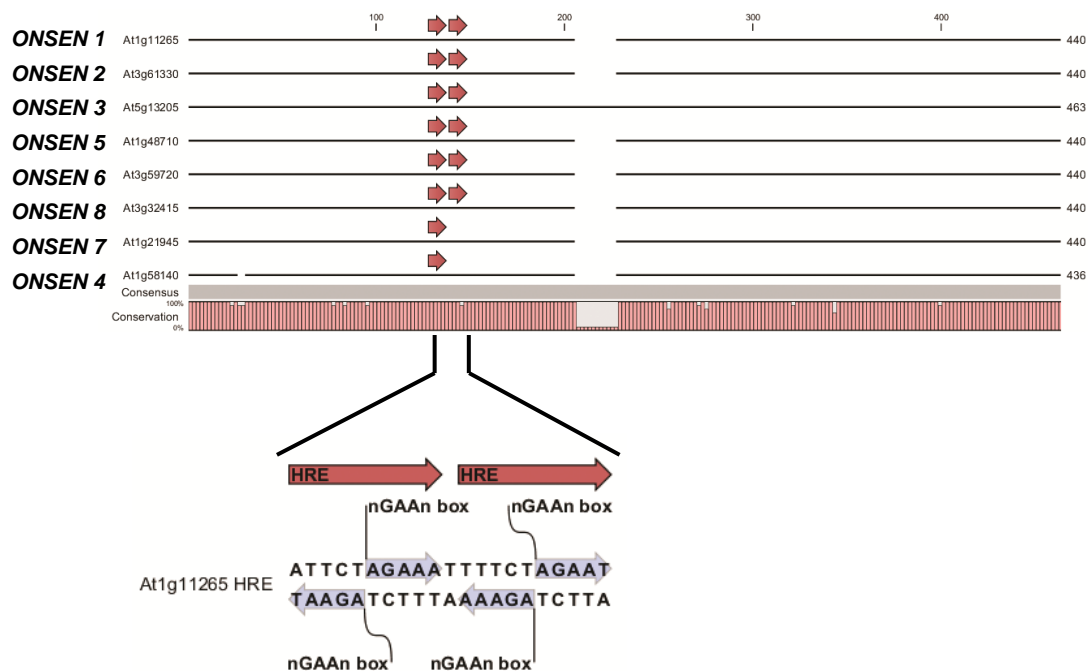

Supplement: Figure S1 — (A) DNA alignment of all 8 ONSEN copies in the Col-0 reference genome. LTRs are indicated in green. The position of the Southern blot probe, the region analyzed for SNPs and the qPCR amplicon are indicated as black boxes. (B) Distribution of potential methylation sites in the LTRs of ONSEN 1 and 2. Note that all cytosines are in the CHH context (green triangles); CG sites or CHG sites are absent. (C) DNA alignment of all 5′LTRs of the 8 ONSEN copies in the Col-0 reference genome. Red arrows indicate heat responsive elements (HRE). nGAAn boxes in these HREs are highlighted with grey arrows. (PDF) [file pgen.1004115.s002.pdf]

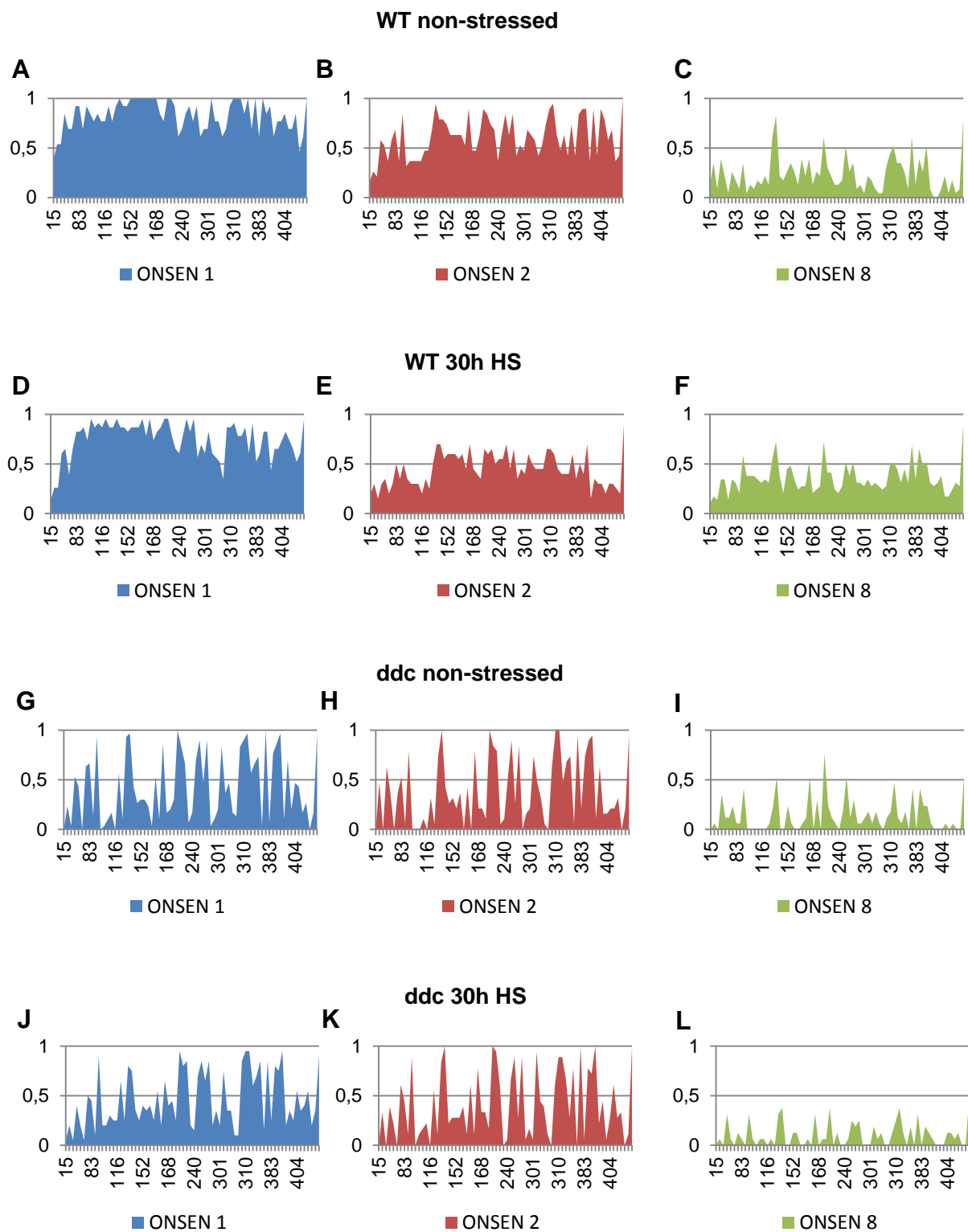

**Cavrak et al. Supplementary Figure 2**

Supplement: Figure S2 — CHH methylation profiles along the LTRs of ONSEN 1 (blue, A,D,G.J) and 2 (red, B,E,H,K) contributing the majority of exDNA and ONSEN 8 (green, C,F,I,L, not activated), in non-stressed (A–C, G–I) and 30 h HS (D–F, J–L) wild type (A–F) and ddc triple mutant (G–L) plants, according to bisulfite sequencing analysis. Only CHH positions shared between all three LTRs were considered. Sequencing data from individual clones are shown as Supplementary Dataset S1. (PDF) [file pgen.1004115.s003.pdf]

Cavrak et al. Supplementary Figure 3

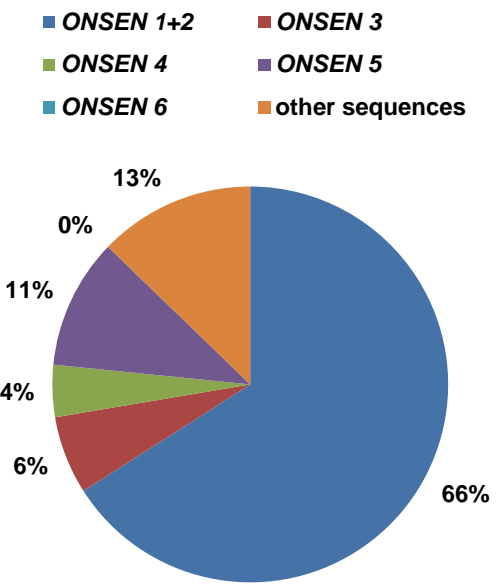

Supplement: Figure S3 — Pie chart indicating the ratio of extrachromosomal ONSEN sequences (n = 47) present after 30 h HS in the triple methylation mutant ddc, distinguished by element-specific polymorphisms (color-coded). Polymorphisms are listed in Supplementary Table S1. (PDF) [file pgen.1004115.s004.pdf]

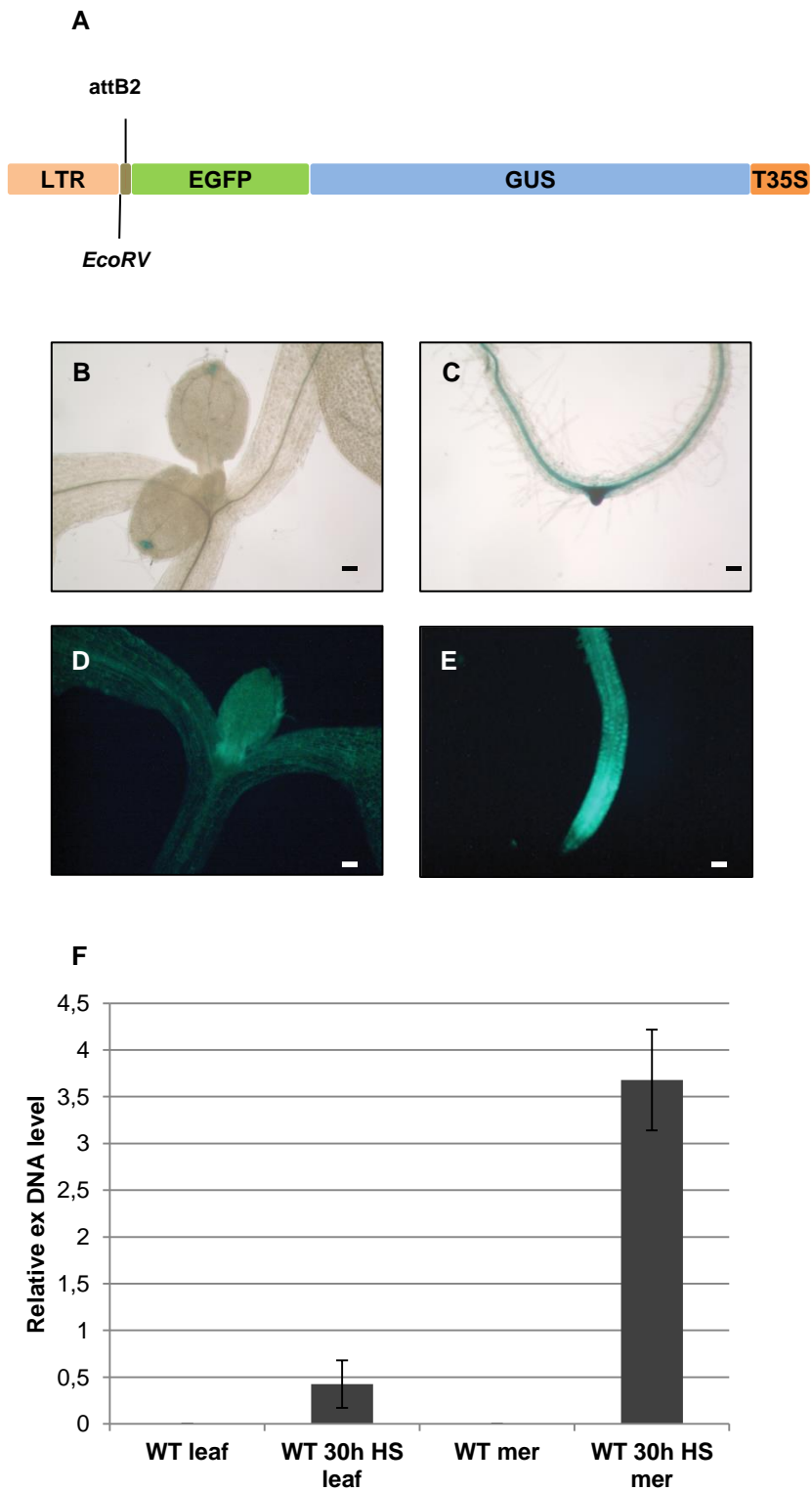

Cavrak et al. Supplementary Figure 4

Supplement: Figure S4 — (A) Schematic representation of the reporter construct with ONSEN promoter (LTR) and visible markers EGFP and GUS in translational fusion. Restriction enzyme EcoRV and Gateway recombination sequence attB2 refer to cloning sites. GUS staining of one week-old seedlings with the GUS reporter gene under control of the ONSEN LTR after 1 h (B) and 2 h heat stress (C). Shoot (D) and root (E) images of one week-old seedlings with the GFP reporter gene under control of the ONSEN LTR after 30 h HS. Scale bars represent 0.1 mm. (F) Quantification of ONSEN extrachromosomal DNA in dissected shoot tips and leaves of three week-old, non-stressed or 30 h HS WT Col-0. Bars represent the ratio between extrachromosomal and integrated copies determined by densitometry after Southern blot analysis. Error bars correspond to the s.d. (n = 2). (PDF) [file pgen.1004115.s005.pdf]
