# Supplementary material for: How a Retrotransposon Exploits the Plant's Heat Stress Response for Its Activation
Source: PLoS Genet. 2014 Jan 30;10(1):e1004115. doi: 10.1371/journal.pgen.1004115 (PMC3907296; doi:10.1371/journal.pgen.1004115)
Supplement: Table S1 — Polymorphisms between ONSEN copies in accession Col-0. (PDF) [file pgen.1004115.s006.pdf]

Cavrak et al. Supplementary Table 1

SNP deviation from At1g48710	Position
C, if G then At1g21945	37
A, if T then At3g59720	42
C, if T then At5g13205	152
T, if C then At3g32415	192
T, if C then At3g32415	195
T, if G then At1g21945	213
C, if A then At3g32415	233
T, if G then At3g32415	247
G, if A then At3g59720	250
A, if C then At3g61330	262
A, if T then At3g32415	279
C, if T then At3g32415	312
G, if A then At3g32415	334
G, if - then At3g32415	386
C, if T then At3g32415	399
C, if T then At3g32415	477
T, if C then At3g32415	486
G, if A then At3g32415	488
T, if C then At3g32415	498
C, if T then At3g32415	548
G, if A then At1g11265 OR At3g61330	331
T, if C then At5g13205	395
C, if T then At1g58140	483
T, if A then At1g21945	516
