# Supplementary material for: How a Retrotransposon Exploits the Plant's Heat Stress Response for Its Activation
Source: PLoS Genet. 2014 Jan 30;10(1):e1004115. doi: 10.1371/journal.pgen.1004115 (PMC3907296; doi:10.1371/journal.pgen.1004115)
Supplement: Table S2 — Oligonucleotide sequences. (PDF) [file pgen.1004115.s007.pdf]

Cavrak et al. Supplementary Table 2

Sequences of oligonucleotides

Gene	Forward primer	Reverse primer	Purpose
ONSEN	CCACAAGAGGAACCAACGAA	TTCGATCATGGAAGACCGG	RT-qPCR
ATSAND	AACTCTATGCAGCATTTGATCCACT	TGATTGCATATCTTTATCGCCATC	RT-qPCR
ONSEN-SNPs	GAAAACGGCCAAAGTTACCA	AGTTGTCCAAGGCTCAAGATG	SNP sequencing
HSFA2	GTGTTGAGGTTGGGCAATACG	CTCCTTAGTAGGCATCGAATTATTCG	Northern probe
At1g11265 ONSEN	AAGAGATATTTAYTGTGAGAT	ATACCACTTTRTTARARTAAA	Bisulfite sequencing
At3g32415 ONSEN	ATATGTGGYAGATYAAYAGA	ATACCACTTTRTTARARTAAA	Bisulfite sequencing
At3g61330 ONSEN	TGGTATAAAGTGTTGTATYTT	ATACCACTTTRTTARARTAAA	Bisulfite sequencing
HSFA2	GTGTTGAGGTTGGGCAATACG	TTGCTGTTGCCTCAACCTAACTAC	RT-qPCR
ONSEN	TGTTGAAAGTTAAACTTGATTTTGAAT	TGTTAGAGTAAAATTCTTTTAGAGGT	LTR for EMSA
ONSEN	CCACCTCCTTAAAAGATTCTAGAAATTTTCTAGAATCATCTTCCACCTCCT	AGGAGGTGGAAGATGATTCTAGAAAATTTCTAGAATCTTTTAAGGAGGTGG	Complete HRE
HSFA2	AGCTCATATGGAAGAACTGAAAGTGGAAATGGAGG	AGCTCTCGAGAGGTTCCGAACCAAGAAAACCCATTTG	Making His-tagged protein
ONSEN	AATACCTCCTCCCCCTCTCA	ATCCTTGATAGATTAGACAGAGAGCT	Southern probe
HSFA2	GTTAGAAATGGGYTTAAGTAAAGG	CCTCCATTTCCACTTTCARTTCTTCC	Bisulfite sequencing
